# CCAR2 controls mitotic progression through spatiotemporal regulation of Aurora B

**DOI:** 10.1038/s41419-022-04990-8

**Published:** 2022-06-07

**Authors:** Jaewook Ryu, Ja-Eun Kim

**Affiliations:** 1 Department of Biomedical Science, Graduate School, Kyung Hee University, Seoul, 02447 Korea; 2 Department of Pharmacology, School of Medicine, Kyung Hee University, Seoul, 02447 Korea

**Keywords:** Mitosis, Checkpoints

## Abstract

CCAR2 (cell cycle and apoptosis regulator 2) is a multifaceted protein involved in cell survival and death following cytotoxic stress. However, little is known about the physiological functions of CCAR2 in regulating cell proliferation in the absence of external stimuli. The present study shows that CCAR2-deficient cells possess multilobulated nuclei, suggesting a defect in cell division. In particular, the duration of mitotic phase was perturbed. This disturbance of mitotic progression resulted from premature loss of cohesion with the centromere, and inactivation of the spindle assembly checkpoint during prometaphase and metaphase. It resulted in the formation of lagging chromosomes during anaphase, leading ultimately to the activation of the abscission checkpoint to halt cytokinesis. The CCAR2-dependent mitotic progression was related to spatiotemporal regulation of active Aurora B. In conclusion, the results suggest that CCAR2 governs mitotic events, including proper chromosome segregation and cytokinetic division, to maintain chromosomal stability.

## Introduction

The genetic and cytoplasmic material in somatic cells must be transmitted equally from a mother cell to two daughter cells during the mitotic phase (M phase is composed of mitosis and subsequent cytokinesis). First, the replicated DNA is condensed into chromosomes, which are segregated during mitosis. Next, cytokinesis separates the cytoplasm to yield two daughter cells, and the physical association is finally severed at the end of cytokinesis. During mitosis and cytokinesis, multiple proteins such as kinases and phosphatases coordinate complexed processes. However, when cells sense errors in mitotic progression, they activate checkpoints that halt the mitotic progression. These checkpoints include the spindle assembly checkpoint (SAC) [1, 2] and the abscission checkpoint [3, 4]. The SAC is a fail-safe mechanism that blocks transition from metaphase-to-anaphase when microtubules do not attach to the kinetochore properly. Until correct binding of microtubule to kinetochore (KT-MT attachment) is achieved, cells delay onset of anaphase by inhibiting anaphase-promoting complex/cyclosome (APC/C). The abscission checkpoint provides cells with time to resolve the problem of trapped chromatin within the cleavage plane. However, the abscission checkpoint is not a fail-safe mechanism because missegregating and lagging chromosomes are frequently damaged during cytokinesis [5].

The chromosomal passenger complex (CPC) is a critical regulator that commonly functions at the spindle assembly and abscission checkpoints during chromosomal and cytoskeletal events associated with cell division [6–9]. At early mitosis, the CPC orchestrates chromosome condensation, chromosome biorientation, correction of erroneous KT-MT attachments, and cohesion protection. At late mitosis, the CPC controls chromosome decondensation, as well as the formation and function of the contractile ring that drives abscission of the two daughter cells. The CPC comprises a localization module and a kinase module, which are linked by the scaffold protein INCENP. The localization module, which comprises the INCENP N-terminus, borealin, and survivin, is responsible for localization of the CPC to the inner centromere from early prophase to before anaphase, to the spindle midzone and equatorial cortex at anaphase, and to the midbody at cytokinesis. The kinase module, which comprises the INCENP C-terminus and Aurora B kinase, is responsible for phosphorylation of Aurora B substrates in the inner and outer kinetochore, the midzone, and the midbody. Dynamic localization of the CPC ensures its spatiotemporal function through Aurora B kinase activity. Delicate regulation of Aurora B activity within the CPC complex is critical for successful mitotic progression [6]. The phosphorylation of Aurora B substrates is involved in chromosome condensation, relocation CPC to centromeres, spindle midzone or equatorial cortex, bundling of central spindles, cleavage furrow ingression, filament assembly, and midbody stabilization [10–12]. In particular, Aurora B is required for activation of SAC and abscission checkpoint [6, 13].

CCAR2 (cell cycle and apoptosis regulator 2; also known as DBC1, deleted in breast cancer 1) is a multifaceted protein that regulates diverse physiological and pathological conditions. It plays a role in cell growth and death, as well as in the DNA damage response, transcription, chromatin remodeling, RNA splicing, circadian rhythms, and metabolism [14, 15]. Some functions are mediated through negative regulation of SIRT1 deacetylase activity, but some functions are SIRT1-independent. With regard to cancer, the role of CCAR2 as either a tumor promoter or suppressor is still controversial. Although CCAR2 governs cell growth and death in response to cytotoxic stress, little is known about how it controls cell proliferation under normal conditions. Mass spectrometry analysis revealed that CCAR2 may form a functional network with multiple proteins involved in chromosome condensation and segregation [16]; however, no direct interaction has been reported. In addition, CCAR2 is required for the expression of a subset of cell cycle-regulated genes in human squamous cell carcinoma cells [17]. A recent report also showed that CCAR2 is required for exit from polyoma small T (PyST)-induced mitotic arrest [18]. Although a role for CCAR2 in regulating mitosis has been suggested, no study has reported the direct role of CCAR2 in mitosis.

The present study shows that CCAR2 deficiency perturbs mitotic progression. Premature loss of cohesion and occurrence of lagging chromosomes is enhanced in CCAR2-deficient cells. Erroneous mitotic progression results ultimately in delayed cytokinesis. Taken together, our data suggest that CCAR2 plays a critical role in ensuring equal partitioning of genomic and cytoplasmic contents to both daughter cells.

## Results

### CCAR2 is required for proper cell proliferation

**Fig. 1:**
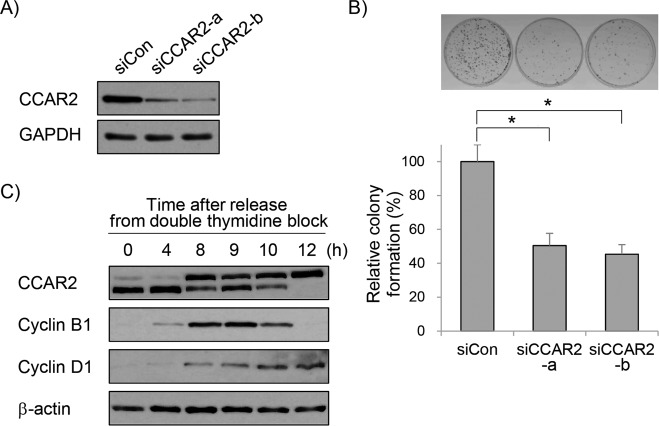
CCAR2 deficiency inhibits cell proliferation. A549 cells were transfected with siRNAs targeting CCAR2. All subsequent assays were done 48 h later. **A** Expression of CCAR2 was verified by western blotting. **B** Cells were seeded to allow colony growth for 14 days. The number of colonies from five separate experiments was counted (*N* = 5). **C** Cells were arrested at the G1/S boundary by double thymidine block and then released to the next phase of cell cycle, which was confirmed by measuring cyclin expression by western blotting. Data are expressed as the mean ± standard error of the mean (SEM). *p < 0.05, significantly different between multiple groups (one-way ANOVA).

The role of CCAR2 during cell proliferation was analyzed by transfecting A549 cells with two different siRNAs. The efficiency of CCAR2 knock-down was determined by measuring levels of protein and mRNA (Fig. 1A and Supplementary Fig. 4A). The effect of CCAR2 deficiency on clonogenic growth was examined in a colony formation assay, and CCAR2-deficient cells (siCCAR2) had significantly lower growth than that of control cells (siCon) (Fig. 1B). However, this low survival was not due to either cell death or cell cycle arrest (Supplementary Fig. 1A−D). To investigate whether CCAR2 is related to cell division, we examined the expression of CCAR2 protein during the cell cycle. Interestingly, as we previously reported [19], an up-shifted band of CCAR2 started to appear at G2/M transition, coinciding with peak expression of cyclin B1 (Fig. 1C). This indicates that CCAR2 expression is regulated during cell cycle. Overall, these findings suggest that CCAR2 plays a role in cell cycle regulation.

### CCAR2 is essential for faithful mitotic progression

**Fig. 2:**
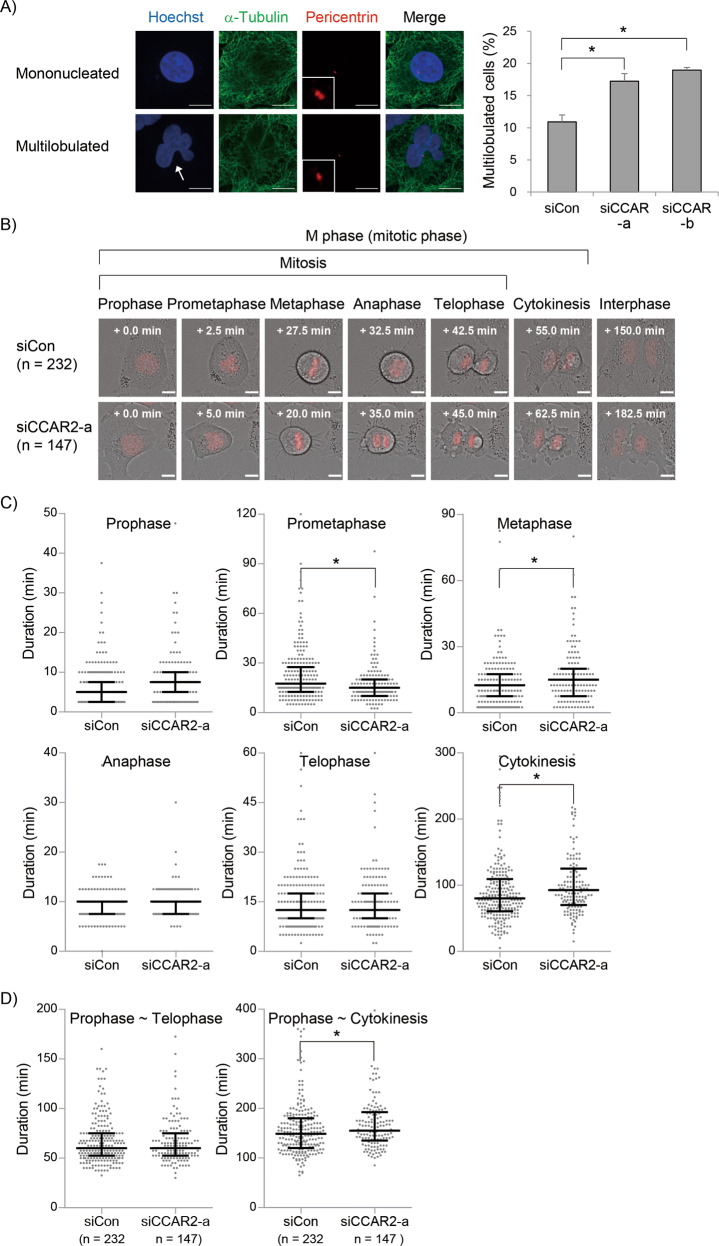
CCAR2 deficiency results in aberrant mitotic progression. **A** Nuclei, the cytoplasm, and the centrosome were visualized by staining with Hoechst, anti-α-tubulin, and anti-pericentrin antibodies, respectively. Arrows indicate multilobulated nuclei. The number of cells containing a multilobulated nucleus was counted from out of more than 250 cells per experiment. The experiment was repeated independently (*N* = 6). The number of interphase cells examined in all experiments is as follows; siCon, *n* = 1620; siCCAR2-a, *n* = 1693; siCCAR2-b, *n* = 1772. Data are expressed as the mean ± SEM. *p < 0.05, significantly different between multiple groups (one-way ANOVA); scale bar, 10 μm. **B** Cells expressing H2B-RFP were subjected to live-cell imaging fluorescence microscopy. Individual cells were tracked for 48 h. The time in the rectangle represents a starting point of each phase. Scale bar, 10 μm. **C** The duration of each mitotic phase is presented as a dot plot. **D** The duration of the entire mitosis (left panel) and M phase (including cytokinesis; right panel) is presented as a dot plot. Live images from three separate experiments (*N* = 3) were analyzed (siCon, *n* = 232; siCCAR2-a, *n* = 147). The first and third bars are the 25th and 75th percentiles, respectively, and the second bar is the median. *p < 0.05, significantly different from corresponding siCon cells (Mann–Whitney U test).

Although the change in CCAR2 expression during cell cycle is not clearly understood, we hypothesized that defects in cell proliferation in siCCAR2 cells were accumulative after cell division. Normally, cell division produces two daughter cells, both of which possess an oval or round nucleus. However, CCAR2 deficiency resulted in the formation of a multilobulated nucleus in A549 and HeLa cancer cells, and IMR-90 and WI-38 normal cells (Fig. 2A and Supplementary Fig. 3B). This suggests that CCAR2 deficiency induces abnormal nuclear division at the end of mitotic phase. Next, the mitotic phase was examined by checking chromosome condensation, alignment, and movement, and membrane scission (Fig. 2B). Both siCon and siCCAR2 cells succeeded in producing two daughter cells, leading to complete cell division. However, the duration of prometaphase was shortened, but that of metaphase and cytokinesis was prolonged in siCCAR2 cells (Fig. 2C). The other analysis of mitotic portion by immunofluorescence staining supported these findings except for the prolonged metaphase although it is not clearly explainable (Supplementary Fig. 3C). The mitotic duration from prophase to telophase was similar between siCon and siCCAR2 cells (Fig. 2D) because the change in prometaphase and metaphases was offset. By contrast, the total duration from prophase to cytokinesis was significantly longer in siCCAR2 cells due to the prolonged cytokinesis (Fig. 2D). It is consistent with data showing the similar level of Histone H3-pS10 which starts to be dephosphorylated at the end of mitosis and finally is not detected in cytokinesis (Supplementary Figs. 1D, 2A). Overall, the defect in cell proliferation shown in Fig. 1 is likely associated with abnormal mitotic progression. This suggests that CCAR2 deficiency presents a hurdle to cell division.

### CCAR2 fine-tunes cohesion release and chromosome condensation

**Fig. 3:**
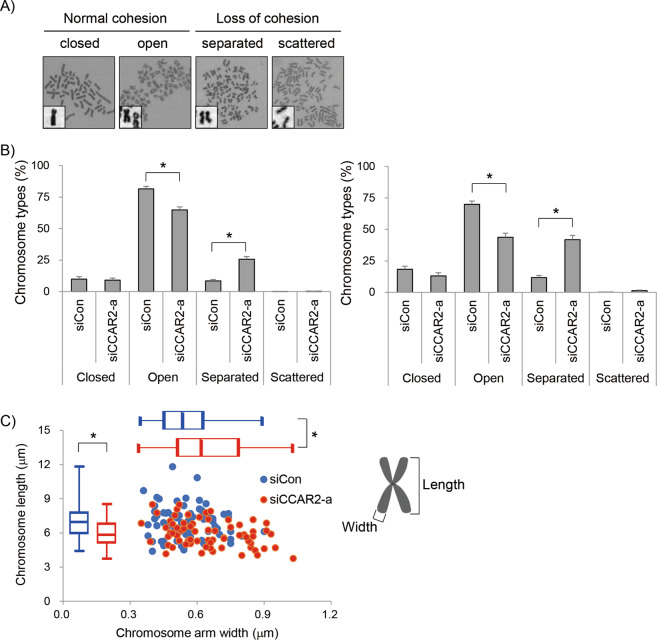
CCAR2 deficiency induces premature sister chromatid separation and abnormal chromosome condensation. **A**−**C** Metaphase spreads were prepared following treatment of cells with 25 ng/ml colcemid for 6 h, or 50 μM monastrol for 3 h followed by release in the presence of 20 μM MG132 for 1.5 h. **A** Cohesion phenotypes were classified into four categories: (i) closed, sister chromatid arms are unresolved; (ii) open, sister chromatid arms are resolved; (iii) separated, sister chromatid arms and centromere are separated, but remain near each other; (iv) scattered, individual sister chromatids are fully scattered. The former two states are normal, but the latter two indicate loss of cohesion. **B** The metaphase spread with each cohesion phenotype was counted from out of more than 20 metaphase spreads per experiment. The experiment was repeated independently (*N* = 7). The total number of metaphase spreads examined is as follows; colcemid-treated cells in left panel, siCon, *n* = 195; siCCAR2-a, *n* = 194; monastrol/MG132-treated cells in right panel, siCon, *n* = 182; siCCAR2-a, *n* = 154. Data are expressed as the mean ± SEM. *p < 0.05, significantly different from corresponding siCon cells (Mann–Whitney U test). **C** Metaphase spreads were prepared following treatment of cells with 50 μM monastrol for 3 h followed by a wash-out in the presence of 20 μM MG132 for 1.5 h. The longest chromosome from each spread (chromosome 1) was analyzed. Ten metaphase spreads per experiment were examined and the experiment was repeated independently (*N* = 7, *n* = 70). The chromosome length and arm width were plotted, and also represented in box-and-whisker plot. Box, interquartile range; whisker, min and max; bar, median. *p < 0.05, significantly different from corresponding siCon cells (two-sided unpaired Student’s t-test).

Aberrant mitotic progression is vulnerable to the accumulation of chromosomal instability. Next, we asked why the transition from prometaphase to metaphase is accelerated. At this stage, microtubules are polymerized to plus ends, which attach to the kinetochore, and the chromosomes continue to condense and become bioriented. In addition, a stepwise loss of cohesion is scheduled from prophase to the end of metaphase. Cohesin, a multisubunit protein complex, is associated with replicated sister chromatids to form cohesion [20]. Cohesion situated close to the centromere and pericentromere should be maintained until onset of anaphase to ensure biorientation of chromosomes and prevents premature chromosome segregation [21–23]. Therefore, to verify whether CCAR2 controls cohesion-dependent chromosome organization, mitotic spreads were prepared from cells arrested at prometaphase and metaphase by treatment with colcemid [24]. Chromosomes with different cohesion status were classified into four categories (Fig. 3A) [25, 26]. Control cells contained mostly open chromosomes with resolved arms but with a cohered centromere. By contrast, CCAR2 deficiency resulted in a significant increase in cells containing separated chromosomes, showing both loss of arm cohesion and centromeric cohesion (Fig. 3B, left panel). Cells were also treated with monastrol, an Eg5 inhibitor, and then released in the presence of MG132, which inhibits onset of anaphase. The mitotic spread obtained from the cells also showed the same pattern, i.e., open and separated chromosomes (Fig. 3B, right panel). The data indicate that CCAR2 is required to maintain cohesion at the centromere. However, scattered chromosomes with complete loss of cohesion were rarely observed in both siCon and siCCAR2 cells. This suggests that siCCAR2 cells succeeded in establishing cohesion during S phase although a slight increase in S phase still implied some problems with cohesion establishment in siCCAR2 cells (Supplementary Fig. 1B) [27]. Overall, CCAR2 deficiency may rather cause premature loss of cohesion, which might contribute to the accelerated progression of prometaphase (Fig. 2C). Cohesin subunits or cohesin-associated proteins at the chromosome arm are also important for chromosome condensation in yeast, and possibly in mammals [22, 28–31]. This prompted us to investigate the compaction of chromosomes. Mitotic spreads were prepared from the arrested cells mainly at metaphase by treatment with monastrol, followed by release in the presence of MG132 to block onset of anaphase. The width and length of the arms in chromosome 1 spread were measured. siCCAR2 cells had wider and shorter chromosomes (Fig. 3C), demonstrating that CCAR2 is required for proper chromosome condensation. Overall, the data suggest that CCAR2 plays a role in cohesion protection and chromosome condensation.

### CCAR2 is required for activation of Aurora B in early mitosis

**Fig. 4:**
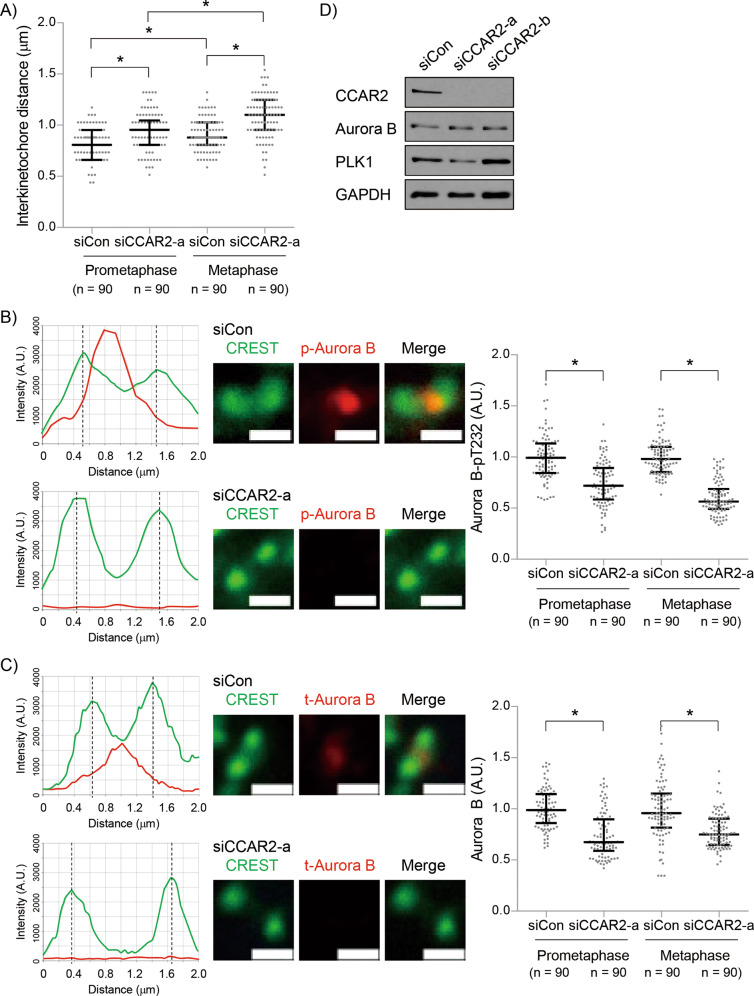
CCAR2 deficiency induces inactivation of Aurora B at prometaphase and metaphase. **A**−**C** Asynchronous cells were stained with Hoechst, CREST, and anti-Aurora B-pT232 or anti-Aurora B antibodies. In total, 90 kinetochores from 30 cells were examined in three separate experiments (*N* = 3, *n* = 90). **A** The distance between kinetochores labeled with CREST was measured at prometaphase and metaphase, and presented as a dot plot. The first and third bars are the 25th and 75th percentiles, respectively, and the second bar is the median. *p < 0.05, significantly different between multiple groups (one-way ANOVA). **B**, **C** Fluorescence intensity of kinetochore-associated Aurora B-pT232 and Aurora B in siCCAR2-a cells was normalized to that of siCon cells at prometaphase and metaphase. *p < 0.05, significantly different from corresponding siCon cells (two-sided unpaired Student’s t-test); A.U., arbitrary units; scale bar, 1 μm. **D** The expression of each protein in asynchronous cells was confirmed by western blotting.

The above data demonstrate that CCAR2 regulates progression of early mitosis through cohesion-dependent chromosome organization. To confirm the loss of cohesion in siCCAR2 cells, the kinetochores were labeled with CREST and the interkinetochore distance between sister kinetochores was measured at prometaphase and metaphase in an asynchronous state. In control cells, the distance was indeed longer at metaphase than at prometaphase due to pulling tension exerted by the microtubules. As expected, CCAR2 deficiency led to increased interkinetochore distance during both phases, indicating that cohesion release at the centromere occurred prematurely in siCCAR2 cells (Fig. 4A). Next, we asked what is responsible for both cohesion protection and chromosome condensation. One of the key molecules during these regulatory processes is Aurora B, a component of the CPC [32, 33]. Therefore, we measured the level of an autophosphorylated Aurora B (Aurora B-pT232), which is indispensable for Aurora B activity. The amount of Aurora B-pT232 at the kinetochore at prometaphase and metaphase in siCCAR2 cells was much lower than that in siCon cells (Fig. 4B). Next, to check whether less Aurora B is recruited, we examined localization of Aurora B at the kinetochore. CCAR2 deficiency also resulted in a defect in Aurora B recruitment (Fig. 4C) although CCAR2 did not affect the expression of Aurora B mRNA and protein (Fig. 4D and Supplementary Figs. 3A, 4A). Instead, the binding between ectopic CCAR2 and Aurora B in HEK293T cells might allow CCAR2-dependent recruitment of Aurora B to kinetochore (Supplementary Fig. 4B). The data suggest that CCAR2 is required for localization and activity of Aurora B, via not its expression but possibly via its interaction, to maintain cohesion protection and chromosome condensation.

### CCAR2 regulates spindle assembly checkpoint

**Fig. 5:**
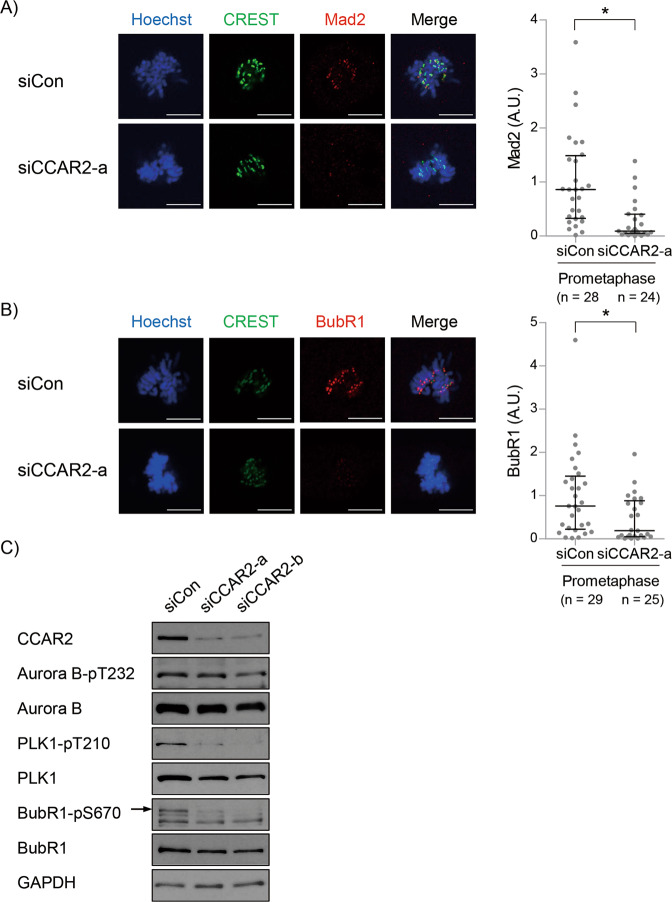
CCAR2 deficiency induces inactivation of spindle assembly checkpoint. **A**, **B** Asynchronous A549 cells were stained with Hoechst, CREST, and anti-Mad2 or anti-BubR1 antibodies. Fluorescence intensity of kinetochore-associated Mad2 (A) or BubR1 (B) in siCCAR2-a cells was normalized to that of siCon at prometaphase. The number of prometaphase in all experiments (*n*) was presented in each graph. The first and third bars are the 25th and 75th percentiles, respectively, and the second bar is the median. *p < 0.05, significantly different from corresponding siCon cells (Mann–Whitney U test); A.U., arbitrary units; scale bar, 10 μm. **C** Cells were arrested at the G1/S boundary by double thymidine block and then released for 9 h. MG132 (30 μM) was treated for the last three hours to block anaphase onset. The amount of each protein was determined by western blotting.

The above data prompted us to investigate the effects of Aurora B inactivation in CCAR2-deficient cells. Inhibiting the activity of Aurora B leads to the accumulation of syntelic and merotelic attachments [34, 35], and failure to recruit SAC-related proteins to the kinetochore [36]. Although the mechanism by which CCAR2 controls the recruitment of Aurora B to kinetochore is unclear, reduced recruitment and activation of Aurora B to kinetochore suggests that CCAR2 deficiency leads to inactivation of the SAC. Indeed, siCCAR2 cells failed to recruit Mad2 and BubR1, both of which are components of mitotic checkpoint component, to the kinetochore (Fig. 5A, B). In addition, PLK1, which is required for a robust SAC [37], was also less recruited and activated in siCCAR2 cells (Supplementary Fig. 5A, B). In prometaphase- and metaphase-enriched cells, we examined the effects of CCAR2 on activation of Aurora B, PLK1, and BubR1 using western blotting. Unexpectedly, in contrast to immunocytochemistry, the levels of total and active Aurora B were not reduced in siCCAR2 cells (Fig. 5C). In fact, the levels of these SAC-related proteins in total cell lysates do not reflect the extent of their recruitment and activation at kinetochore. It means that CCAR2 is not required for partial activation before Aurora B is recruited to kinetochore, but is involved in its recruitment and full activation at kinetochore. By contrast, siCCAR2 cells showed lower levels of PLK1-pT210 and BubR1-pS670, which are dependent on the activity of Aurora B at kinetochore [38, 39] (Fig. 5C). It suggests that a defect in Aurora B functions leads to mitotic slippage, a type of exit from mitotic arrest, through premature inactivation of SAC in siCCAR2 cells.

### CCAR2 is required for proper chromosome segregation

**Fig. 6:**
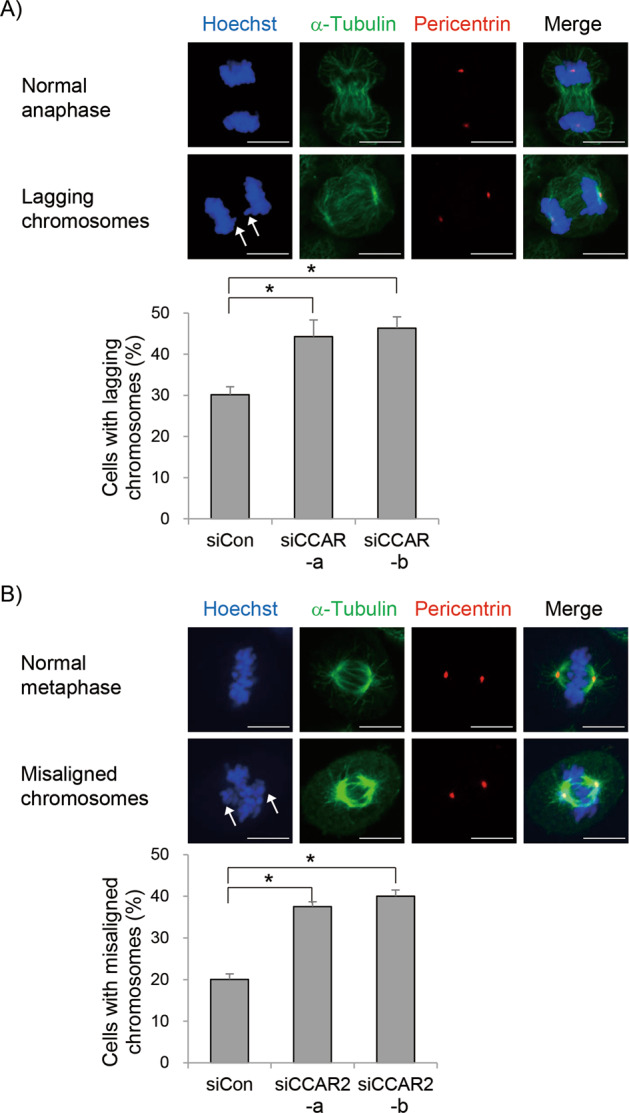
CCAR2 deficiency induces misaligned and lagging chromosomes. **A** Asynchronous cells were stained with Hoechst, anti-α-tubulin and anti-pericentrin antibodies. The number of cells containing lagging chromosomes at anaphase was counted from out of more than 20 anaphase cells per experiment. The experiment was repeated independently (*N* = 6). The number of anaphase examined in all experiments was as follows; siCon, *n* = 140; siCCAR2-a, *n* = 142; siCCAR2-b, *n* = 138. **B** Cells were treated for 3 h with 50 μM monastrol. Arrested cells with monopolar spindles were washed out to allow recovery for 1.5 h in the presence of 20 μM MG132. The number of cells containing misaligned chromosomes at metaphase was counted from out of more than 30 metaphase per experiment. The experiment was repeated independently (*N* = 3). The number of metaphase examined in all experiments was as follows; siCon, *n* = 130; siCCAR2-a, *n* = 121; siCCAR2-b, *n* = 128. Data are expressed as the mean ± SEM. *p < 0.05, significantly different between multiple groups (one-way ANOVA); scale bar, 10 μm.

The SAC is essential for proper chromosome segregation, a process in which two sister chromatids separate and migrate to opposite poles at anaphase [40]. Unless chromosome segregation is faithful, chromosomal instabilities accumulate. The footprint of chromosome segregation errors includes lagging chromosomes, which lag behind the moving chromosome mass at anaphase. We found an increase in the number of lagging chromosome-containing cells in CCAR2-deficient A549, HeLa, IMR-90, and WI-38 cells (Fig. 6A and Supplementary Fig. 6A). One major cause of lagging chromosomes is merotelic attachment, in which a kinetochore of a single chromatid is attached to microtubules emanating from two opposite spindle poles [41–43]. To investigate whether CCAR2 deficiency aggravates the formation of merotelic kinetochore attachments, we performed an error correction assay by treating cells with monastrol, which accumulates syntelic KT-MT attachments before washing-out and increases merotelic attachments after washing-out [44]. Then, cells were maintained at metaphase by incubation with MG132 to block anaphase onset. If they contain the proper machinery to correct attachment errors, cells align chromosomes along the spindle equator. However, siCCAR2 cells showed a higher number of cells with chromosome misalignment at metaphase (Fig. 6B). By contrast, we did not observe any obvious defect in chromosome alignment at metaphase under asynchronous conditions (data not shown). This might be due to the very transient status of merotelic attachments during metaphase. Overall, this demonstrates that CCAR2 ensures proper SAC activation, correction of the KT-MT attachment, and chromosome segregation.

### CCAR2 is required for spatiotemporal regulation of Aurora B to ensure ingression and abscission during late mitosis

**Fig. 7:**
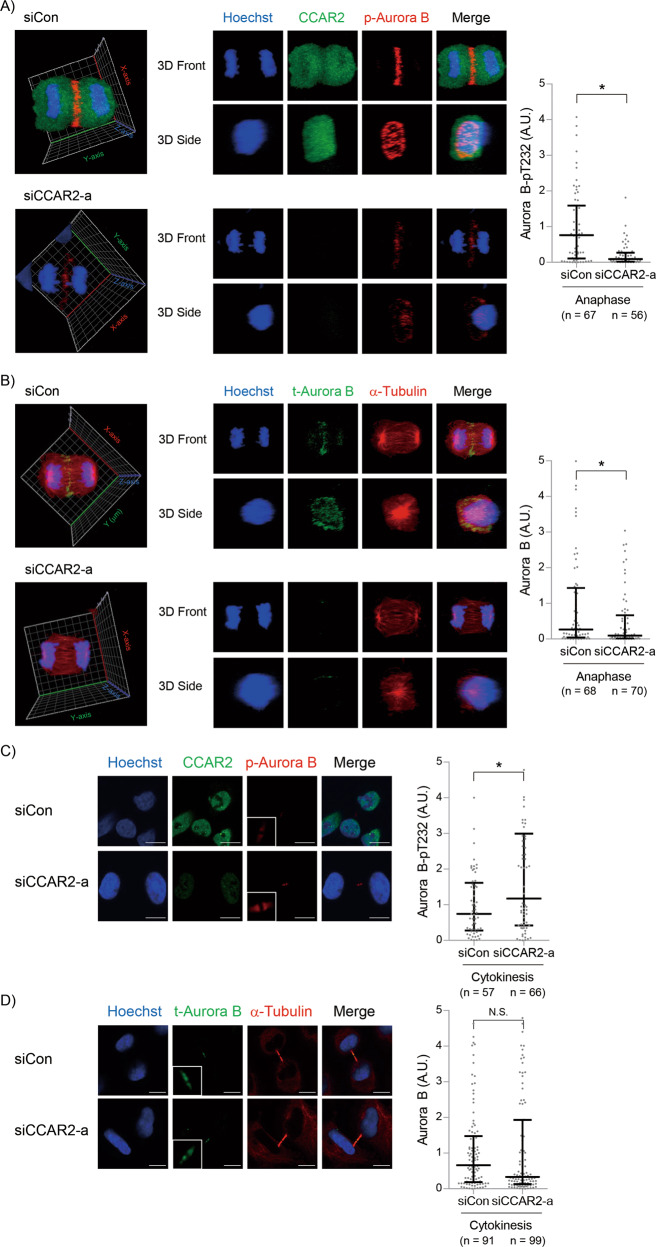
CCAR2 deficiency disrupts the spatiotemporal regulation of Aurora B during late mitosis. Asynchronous cells were stained with Hoechst, and anti-CCAR2, anti-Aurora B-pT232, anti-Aurora B, or anti-α-tubulin antibodies. The levels of Aurora B-pT232 or total Aurora B at the spindle midzone and equatorial cortex during anaphase (**A**, **B**), and at midbody during cytokinesis (**C**, **D**), were examined by measuring fluorescence intensity. The boxes at the corner represent the enlarged midbody. Fluorescence intensity of siCCAR2-a cells was normalized to that of siCon cells at anaphase and cytokinesis. The experiment was repeated independently (*N* = 4). The number of anaphase or cytokinesis in all experiments (*n*) was presented in each graph. The first and third bars are the 25th and 75th percentiles, respectively, and the second bar is the median. *p < 0.05, significantly different from corresponding siCon cells; N.S., not significant (Mann–Whitney U test); A.U., arbitrary units; each grid, 3 μm (Fig. 7A, B); scale bar, 10 μm (Fig. 7C, D).

**Fig. 8:**
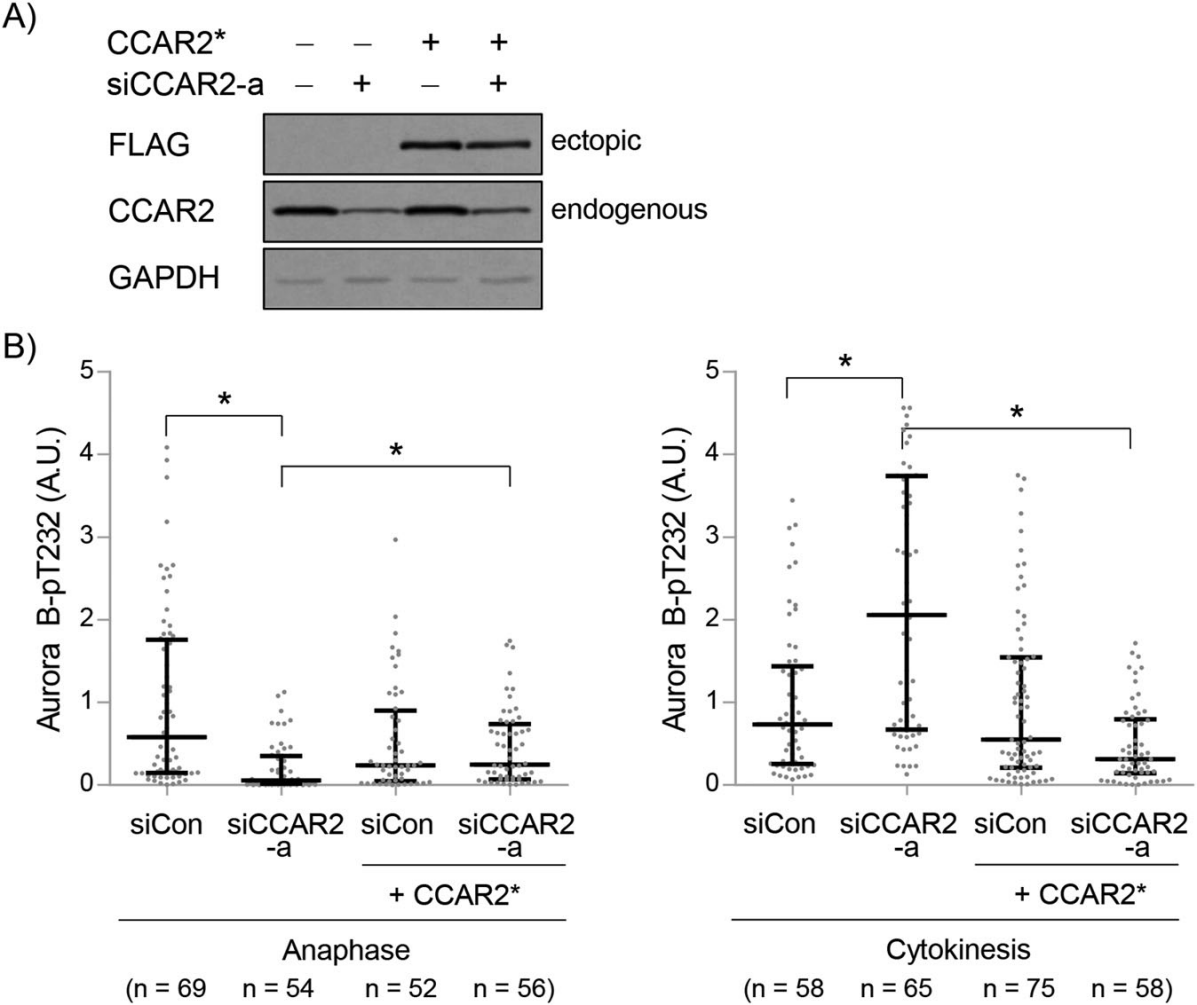
CCAR2 is required for proper regulation of Aurora B during late mitosis. Cells were infected with siRNA-resistant FLAG-CCAR2(*) and then transfected with CCAR2 siRNA-a. **A** Expression of ectopic and endogenous CCAR2 was determined by western blotting. **B** Expression of CCAR2 was verified by immunostaining. The amount of Aurora B-pT232 at the spindle midzone and equatorial cortex during anaphase (left panel), and at midbody during cytokinesis (right panel) were determined by measuring fluorescence intensity. The experiment was repeated independently (*N* = 4). The number of anaphase or cytokinesis in all experiments (*n*) was presented in each graph. The first and third bars are the 25th and 75th percentiles, respectively, and the second bar is the median. *p < 0.05, significantly different between each group (Kruskal–Wallis test); A.U., arbitrary units.

Next, we asked what the final outcome of mitotic defects is induced by CCAR2 deficiencies such as the erroneous KT-MT attachment and lagging chromosomes. Although Aurora B and other SAC-related proteins are partially activated by CCAR2 deficiency at prometaphase or metaphase (Figs. 4B−C, 5A−C), cells were able to undergo onset of anaphase. Then, we observed the activation status of Aurora B during late mitosis. First, the level of Aurora B-pT232 and Aurora B was examined at late anaphase, when the actomyosin contractile ring creates a cleavage furrow. There was markedly less Aurora B-pT232 and Aurora B at the spindle midzone and equatorial cortex in siCCAR2 cells (Fig. 7A, B). This indicates that CCAR2 deficiency leads to mislocalization and inactivation of Aurora B, and possibly inhibits contraction of the cleavage furrow. In addition, the lagging chromosomes observed at anaphase can be a cause of cleavage furrow regression, chromatin cleavage, and/or abscission delay [5]. We ruled out the first two because the number of cells undergoing regression and showing DNA damage was not significant when detected by live imaging and staining of γ-H2AX, a marker of DNA damage (data not shown). The possibility of abscission delay was raised by the observed prolongation of cytokinesis by live imaging (Fig. 2B−D). The Aurora B-dependent abscission checkpoint helps to resolve the anaphase bridge [45, 46]. Therefore, we examined the levels of Aurora B-pT232 at the midbody during cytokinesis, when intercellular bridge severing (known as abscission) occurs. Aurora B-pT232 was enriched significantly at the midbody in siCCAR2 cells (Fig. 7C), suggesting that sustained Aurora B activation triggers the abscission checkpoint. However, the recruitment of Aurora B to midbody was not significantly changed in siCCAR2 cells (Fig. 7D). It suggests that CCAR2 is required for the recruitment of Aurora B to kinetochore, spindle midzone, and equatorial cortex, but not to midbody. Considering that enrichment of microtubules at midzone during anaphase and at midbody during cytokinesis was not affected by CCAR2 deficiency (Supplementary Fig. 7A, B), the major cause for a delay in cytokinesis is prolonged activation of the abscission checkpoint. To rule out any off-target effect of CCAR2 siRNA, siCCAR2 cells were reconstituted with an siRNA-resistant CCAR2-overexpressing construct (Fig. 8A). The low level of Aurora B-pT232 in siCCAR2 cells was rescued by CCAR2 overexpression at the spindle midzone and equatorial cortex during anaphase (Fig. 8B, left panel, group 2 vs. group 4). In addition, the high level of Aurora B-pT232 in siCCAR2 cells was diminished by CCAR2 overexpression at midbody during cytokinesis (Fig. 8B, right panel, group 2 vs. group 4). This demonstrates that CCAR2 specifically controls the spatiotemporal activation of Aurora B.

## Discussion

Mitotic progression is a complex program controlled by mitotic structures, including the chromosome, cohesion, kinetochore, centrosome, central spindle, and midbody, and multiple kinases such as Aurora kinase and Polo-like kinase. Here, we show that CCAR2 is necessary for time-order transition of each mitotic phase by regulating mitotic structures and kinases. CCAR2 controls cohesion dynamics and chromosome condensation, and spatiotemporally coordinates the localization and activity of Aurora B, a component of the CPC. In particular, inactivation of Aurora B by CCAR2 deficiency fails to correct erroneous KT-MT attachment, leading ultimately to chromosomal missegregation. The presence of lagging chromosomes halts cytokinetic division through sustained activation of Aurora B during cytokinesis. Taken together, the data show that CCAR2 is responsible for orchestrating the faithful division of chromosomes and cytoplasm.

Our study demonstrates that CCAR2 is one of the regulatory proteins for mitotic progression. Sarwar et al. showed that CCAR2 is localized to nucleoplasm during interphase, to spindle poles during prometaphase and metaphase, to midzone during anaphase, and midbody during telophase and cytokinesis [18]. However, we observed that CCAR2 is leaked to cytoplasmic region after nuclear envelope breakdown and re-localized in nucleoplasm after nuclear membrane assembly during mitotic phase. In addition, Sarwar et al. presented that CCAR2 expression peaks at G2/M phase. However, the present study shows that CCAR2 with a mobility shift appears at G2/M phase, suggesting a potential post-tanslationally modified CCAR2. While the difference between Sarwar et al.’s and our studies may be due to different antibodies, both suggest that CCAR2 plays a role in mitosis.

Although we did not investigate the removal of cohesion from the arms and centromere independently [47, 48], the higher incidence of separated chromosomes in CCAR2-deficient cells suggests that dissolution of cohesion from the centromere is accelerated by CCAR2 deficiency (Fig. 3B). Therefore, it raises the possibility that premature loss of cohesion might delay metaphase (Fig. 2C) because cells would pause until KT-MT attachment is corrected and tension is fully established [49, 50]. In addition, unscheduled loss of cohesion results in random chromosome segregation and aneuploidy due to Aurora B’s activity at the SAC [51, 52]. However, we did not observe any significant aneuploid phenotypes CCAR2-deficient cells. The possible explanation for this is that inactivation of the SAC permits mitotic defects in the presence of premature cohesion loss. In fact, the low activity of Aurora B following CCAR2 deficiency allowed mitotic segregation.

Next, we asked what is the mechanism by which CCAR2 deficiency leads to reduced chromosome compaction. It is widely accepted that proper timing of cohesion release from the chromosome arms may be prerequisite for chromosome condensation [20, 53]. Considering the increase in separated chromosomes in CCAR2-deficient cells, premature loss of cohesion might result in insufficient loading of condensin in CCAR2-deficient cells. Condensin II is important for axial shortening (length) of chromosomes [54, 55], but condensin I is extremely important for lateral compaction (width) of chromosomes [56, 57]. Indeed, a previous report also shows that condensin-I-depleted cells possess shorter and wider chromosomes [57]. Given that CCAR2-deficient cells had shorter and wider chromosomes (Fig. 3C), there might be a defect in the function of condensin I. In addition, while depletion of condensin II does not affect the interkinetochore distance, depletion of condensin I increases the interkinetochore distance in metaphase chromosomes, resulting in the failure of centromere tethering [58]. This is consistent with our data showing an increased interkinetochore distance in CCAR2-deficient cells (Fig. 4A), suggesting that CCAR2 is involved in the functions of condensin I rather than condensin II. Taken together, our data suggest that CCAR2 is required for temporal regulation of cohesion maintenance and chromosome condensation.

The present study suggests that CCAR2 mainly regulates the spatiotemporal activity of Aurora B. However, Best et al. showed that CCAR2 deficiency inhibits transcription of a subset of mitotic genes including AURKB, in various squamous cell carcinoma-derived cell lines, possibly through downregulation of the RFX1 and CREB transcription factors [17]. In contrast to previous reports, we found that CCAR2 does not affect the expression of its mRNA or protein in lung-derived cell lines (Fig. 4D and Supplementary Figs. 3A, 4A). Instead, CCAR2 interacts with Aurora B (Supplementary Fig. 4B), suggesting that CCAR2 is a regulatory protein for its recruitment and the following activation. However, it is still unclear how CCAR2 spatiotemporally controls recruitment of Aurora B or Aurora B-associated CPC to a proper mitotic structure.

Aurora B participates in protection of centromeric cohesion [32, 59] and condensin I-dependent chromosome condensation [60, 61]. Aurora B is also a principal regulator of KT-MT detachment through phosphorylation of diverse substrates such as the KMN network [62, 63]. Therefore, Aurora B activity is important for preventing premature removal of SAC proteins from kinetochores before correction of KT-MT attachment [6]. Considering the low activity of Aurora B in the CREST-labeled kinetochore of CCAR2-deficient cells, CCAR2 is required for chromosome maintenance during early mitosis. The low activity of Aurora B in the spindle midzone and equatorial cortex of CCAR2-deficient cells during anaphase might lead to a problem with ingression of the cleavage furrow due to reduced contractile force. By contrast, surprisingly, the high activity of Aurora B contributes to prolonged cytokinesis through activation of the abscission checkpoint. The detailed mechanism by which Aurora B activity is reversed from early mitosis to cytokinesis in CCAR2-deficient cells is not understood. However, our study suggests that CCAR2 participates in tight spatiotemporal regulation of Aurora B to ensure faithful chromosome segregation and cytokinesis. Finally, the dysregulation of Aurora B following CCAR2 deficiency might produce overall defects in cohesion release, chromosome condensation, SAC, ingression of the cleavage furrow, and cytokinesis [7, 63, 64]. Overall, our study suggests that CCAR2 is a factor that fine-tunes Aurora B in mitotic progression.

## Materials and methods

### Cell lines

A549 non-small cell lung cancer cells, HeLa cervical adenocarcinoma cells, IMR-90 and WI-38 normal lung fibroblasts, and HEK293T cells were obtained from the American Type Culture Collection. Cells were maintained in Dulbecco’s modified Eagle’s medium (Welgene Inc.; LM001-05) or Eagle’s Minimum Essential Medium (Welgene Inc.; LM007-07) supplemented with 10% fetal bovine serum, 100 U/ml penicillin G sodium, 100 μg/ml streptomycin sulfate, and 0.25 μg/ml amphotericin B. Cells were incubated at 37 °C in a 5% CO_2_ incubator. The cell lines were routinely tested for the presence of mycoplasma with Hoechst staining.

### Transfection and infection

Control and CCAR2 small interfering RNA (siRNAs) were synthesized by ST Pharm. Co., LTD. The siRNA duplexes were as follows: control siRNA, AUGAACGUGAAUUGCUCAAdTdT; CCAR2 (NM_021174) siRNA-a, CCAUAAUUCUUGCCUCUUUdTdT; CCAR2 siRNA-b, CAGCGGGUCUUCACUGGUAdTdT. In the case that siRNA name is not listed in Results and Figure legends, we used CCAR2 siRNA-a. Forty-eight hours after transfection, all experiments were performed. For reconstitution of CCAR2 in CCAR2 siRNA-transfected cells, cells were first infected with retrovirus encoding Flag-tagged and siRNA-resistant CCAR2 in the presence of polybrene (8 μg/ml). Six hours after infection, the cells were transfected with CCAR2 siRNA. Transfection was performed with 20 nM siRNA using Lipofectamine RNAiMax (Invitrogen; 13778150).

### Clonogenic assay

Cells (1 × 10^3^ cells per 60 mm dish) were plated and then incubated for 14 days. After removal of the medium, cells were rinsed with phosphate-buffered saline (PBS), fixed in acetic acid:methanol (1:7 vol/vol) at room temperature for 5 min, and then stained with staining solution (0.5% crystal violet in 25% methanol). Colonies were counted on triplicate dishes, and independent experiments were repeatedly done.

### Cell synchronization

The G1/S synchronization was achieved by double thymidine block. Cells were cultured in the presence of 2 mM thymidine for 19 h and then released to grow for 10 h. Cells were then treated for another 15 h with 2 mM thymidine, causing cells to arrest at the G1/S boundary. The arrested cells were allowed to enter the S phase by washing-off the thymidine with PBS. Then, cells were released for 9 h in normal culture medium and incubated with 30 μM MG132 for the last 3 h. The mitotic cells were enriched prior to anaphase onset.

### Preparation of crude cell extracts and western blotting

Cells were lysed on ice for 10 min using NETN lysis buffer (100 mM NaCl, 1 mM EDTA, 20 mM Tris-HCl, 0.5% Nonidet P-40, 50 mM β-glycerophosphate, 10 mM NaF, and 1 mM Na_3_VO_4_) containing a protease inhibitor cocktail (Millipore; 535140). After centrifugation at 12,000 × *g* for 5 min, the supernatant was saved as a crude cell extract. This was boiled in Laemmli buffer and loaded onto SDS-polyacrylamide gel. Western blotting was performed according to a standard protocol. The following antibodies were used for western blotting: GAPDH (Santa Cruz Biotechnology; sc-25778), β-actin (Cell Signaling Technology, 4970), FLAG (Sigma; 3165), Myc (Cell Signaling Technology; 2278), Cyclin B1 (Santa Cruz Biotechnology; sc-752), Cyclin D1 (Santa Cruz Biotechnology; sc-8396), Aurora B (Cell Signaling Technology, 3094), Aurora B-pT232 (Santa Cruz Biotechnology; sc-293127), PLK1 (Santa Cruz Biotechnology; sc-55504), PLK1-pT210 (Abcam, ab39068), BubR1 (BD Biosciences, 612503). BubR1-pS670 antibody was provided by C.W. Lee (Sungkyunkwan University). CCAR2 antibody was obtained from immunized rabbit with GST-fused CCAR2 recombinant protein [65, 66].

### Immunocytochemical fluorescence staining

The cells grown on coverslips were fixed with 3% paraformaldehyde solution at room temperature for 10 min and then permeabilized with 0.5% Triton X-100 at room temperature for 5 min. The cells were incubated with antibody against Aurora B (Santa Cruz Biotechnology; sc-25426), Aurora B-pT232 (Santa Cruz Biotechnology; sc-293127), PLK1 (Santa Cruz Biotechnology; sc-55504), PLK1-pT210 (Abcam; ab39068), Mad2 (Pierce; PA5-21594), BubR1 (BD Biosciences; 612503), histone H3-pS10 (Sigma; 06570), CCAR2 (Bethyl Laboratories; A300-434A), CREST (ImmunoVision, Springdale; HCT-0100), Pericentrin (Abcam; ab28144) or α-Tubulin (Invitrogen; PA5-29444, Sigma; T5168) at 37 °C for 20 min and then incubated with corresponding secondary antibody at 37 °C for 20 min. The nuclei were counterstained with Hoechst 33342 (Invitrogen; H21492). After a final wash with PBS, coverslips were mounted with antifade solution containing para-phenylenediamine and glycerol in PBS.

### Live-cell imaging

Cells were infected with a lentivirus encoding histone H2B-RFP in the presence of polybrene (8 μg/ml). Time-lapse imaging was then performed using a fluorescence microscope (Nikon Corporation; ECLIPSE Ti2-E) equipped with a top-stage incubator system (LCI; TC-W, FC-5N). Frames were recorded every 2.5 min. Cell morphology was visualized under a differential interference contrast microscope, and red fluorescence representing histone H2B was detected to track chromosome condensation and movement.

### 3D image acquisition

Three-dimensional image stacks of mitotic cells were acquired under a laser-scanning confocal microscope (Carl Zeiss; LSM700) or an inverted microscope (Nikon Corporation; ECLIPSE Ti2-E). The z-stacks were scanned with a step size of 0.38 µm or 0.2 µm.

### Error correction assay

To induce the formation of errors in KT-MT attachment, cells were incubated for 3 h with 50 μM monastrol and then washed out for 1.5 h into normal culture medium containing 20 μM MG132. By blocking onset of anaphase, the cells allow the checkpoint machinery to correct erroneous attachments [25, 67]. The number of cells containing misaligned chromosomes at metaphase was counted.

### Analysis of mitotic cells

Stained cells were observed under an inverted microscope (Nikon Corporation; ECLIPSE Ti2-E). Cells were selected randomly and the counting was done in a blinded manner. The multilobulated cells were determined based on nuclear membrane morphology. The misaligned chromosomes at metaphase and lagging chromosomes at anaphase were determined based on chromosome alignment [68].

### Preparation of mitotic spread

Chromosomes were prepared as follows [69]. Cells were treated with 25 ng/ml of colcemid, a synthetic analog of colchicine (Invitrogen; 15212012) for 6 h, or treated with 50 μM monastrol for 3 h and then released to the next mitotic step in the presence of 20 μM MG132 for 1.5 h at 37 °C in 5% (v/v) CO_2_ incubator. Cells in medium were collected by mitotic shake-off and the attached cells were harvested by trypsinization. Cells were harvested by centrifugation at 1000 rpm for 4 min. All but 0.5 ml of medium was aspirated and gently used to resuspend cells. The harvested cells were swollen in 5 ml of hypotonic solution (0.075 M KCl) to produce osmotic gradient at 37 °C for 30 min. The fragile cells were collected by spinning down at 1000 rpm for 4 min. All but 0.5 ml of supernatant was aspirated and gently used to resuspend cells. One milliliter of freshly prepared Carnoy’s fixative (methanol:acetic acid = 3:1 vol/vol) was added by dropping while agitating. Cells were incubated at room temperature for 10 min. After subsequent centrifugation at 1000 rpm for 4 min, pellets were resuspended in 3 ml Carnoy’s fixative. This fixation was repeated one more time. After resuspending pellets in a small volume of Carnoy’s fixative, the mitotic spreads were prepared by dropping the fixed swollen cells onto glass slides to release chromosomes through ruptured cell membrane. After air-dry, slides were soaked in Giemsa staining solution. The number and morphology of chromosomes were analyzed under the microscope.

### Data and statistical analysis

All assays were repeated more than three times. Statistical analysis was performed using SPSS software (IBM; version 23). Differences between two groups were evaluated using an unpaired Student’s t-test (parametric analysis) or the Mann–Whitney U test (non-parametric analysis). Differences between three or more groups were evaluated using one-way analysis of variance (ANOVA) followed by Tukey’s honest significant difference (HSD) (parametric analysis) or using the Kruskal–Wallis test followed by Dunn’s multiple comparison test (non-parametric analysis). Post hoc tests were run only if F-test achieved *P* < 0.05 and there was no significant inhomogeneity. Statistical differences were considered significant at *P* < 0.05, and are indicated by asterisk (*).

## Supplementary information

Supplementary Information

Original Data File

Checklist

## Data Availability

All data needed to evaluate the conclusions in the paper are present in the paper. Original data of western blotting were already evaluated by reviewers and others can be available upon reasonable request.

## References

[1] Lara-Gonzalez P, Westhorpe FG, Taylor SS (2012). The spindle assembly checkpoint. Curr Biol.

[2] Musacchio A (2015). The molecular biology of spindle assembly checkpoint signaling dynamics. Curr Biol.

[3] Petsalaki E, Zachos G (2019). Building bridges between chromosomes: Novel insights into the abscission checkpoint. Cell Mol Life Sci.

[4] Mierzwa B, Gerlich DW (2014). Cytokinetic abscission: Molecular mechanisms and temporal control. Dev Cell.

[5] Ganem NJ, Pellman D (2012). Linking abnormal mitosis to the acquisition of DNA damage. J Cell Biol.

[6] Carmena M, Wheelock M, Funabiki H, Earnshaw WC (2012). The chromosomal passenger complex (CPC): From easy rider to the godfather of mitosis. Nat Rev Mol Cell Biol.

[7] Kitagawa M, Lee SH (2015). The chromosomal passenger complex (CPC) as a key orchestrator of orderly mitotic exit and cytokinesis. Front Cell Dev Biol.

[8] van der Horst A, Lens SM (2014). Cell division: Control of the chromosomal passenger complex in time and space. Chromosoma.

[9] Trivedi P, Stukenberg PT (2020). A condensed view of the chromosome passenger complex. Trends Cell Biol.

[10] Kitagawa M, Fung SY, Onishi N, Saya H, Lee SH (2013). Targeting Aurora B to the equatorial cortex by MKlp2 is required for cytokinesis. PLoS One.

[11] Adriaans IE, Hooikaas PJ, Aher A, Vromans MJM, van Es RM, Grigoriev I (2020). MKLP2 is a motile kinesin that transports the chromosomal passenger complex during anaphase. Curr Biol.

[12] Babkoff A, Cohen-Kfir E, Aharon H, Ravid S (2021). Aurora-B phosphorylates the myosin II heavy chain to promote cytokinesis. J Biol Chem.

[13] Vader G, Maia AF, Lens SM (2008). The chromosomal passenger complex and the spindle assembly checkpoint: Kinetochore-microtubule error correction and beyond. Cell Div.

[14] Kim JE, Chen J, Lou Z (2009). p30 DBC is a potential regulator of tumorigenesis. Cell Cycle.

[15] Magni M, Buscemi G, Zannini L (2018). Cell cycle and apoptosis regulator 2 at the interface between DNA damage response and cell physiology. Mutat Res.

[16] Giguere SS, Guise AJ, Jean Beltran PM, Joshi PM, Greco TM, Quach OL (2016). The proteomic profile of deleted in breast cancer 1 (DBC1) interactions points to a multifaceted regulation of gene expression. Mol Cell Proteom.

[17] Best SA, Nwaobasi AN, Schmults CD, Ramsey MR (2017). CCAR2 is required for proliferation and tumor maintenance in human squamous cell carcinoma. J Invest Dermatol.

[18] Sarwar Z, Nabi N, Bhat SA, Gillani SQ, Reshi I, Un Nisa M (2022). Interaction of DBC1 with polyoma small T antigen promotes its degradation and negatively regulates tumorigenesis. J Biol Chem.

[19] Kim JE, Sung S (2010). Deleted in breast cancer 1 (DBC1) is a dynamically regulated protein. Neoplasma.

[20] Peters JM, Tedeschi A, Schmitz J (2008). The cohesin complex and its roles in chromosome biology. Genes Dev.

[21] Tanaka T, Fuchs J, Loidl J, Nasmyth K (2000). Cohesin ensures bipolar attachment of microtubules to sister centromeres and resists their precocious separation. Nat Cell Biol.

[22] Gerton J (2005). Chromosome cohesion: A cycle of holding together and falling apart. PLoS Biol.

[23] Ng TM, Waples WG, Lavoie BD, Biggins S (2009). Pericentromeric sister chromatid cohesion promotes kinetochore biorientation. Mol Biol Cell.

[24] Wiley JE, Sargent LM, Inhorn SL, Meisner LF (1984). Comparison of prometaphase chromosome techniques with emphasis on the role of colcemid. Vitro.

[25] Wike CL, Graves HK, Hawkins R, Gibson MD, Ferdinand MB, Zhang T (2016). Aurora-A mediated histone H3 phosphorylation of threonine 118 controls condensin I and cohesin occupancy in mitosis. Elife.

[26] Alomer RM, da Silva EML, Chen J, Piekarz KM, McDonald K, Sansam CG (2017). Esco1 and Esco2 regulate distinct cohesin functions during cell cycle progression. Proc Natl Acad Sci USA.

[27] Guillou E, Ibarra A, Coulon V, Casado-Vela J, Rico D, Casal I (2010). Cohesin organizes chromatin loops at DNA replication factories. Genes Dev.

[28] Tedeschi A, Wutz G, Huet S, Jaritz M, Wuensche A, Schirghuber E (2013). Wapl is an essential regulator of chromatin structure and chromosome segregation. Nature.

[29] Lopez-Serra L, Lengronne A, Borges V, Kelly G, Uhlmann F (2013). Budding yeast Wapl controls sister chromatid cohesion maintenance and chromosome condensation. Curr Biol.

[30] Guacci V, Koshland D, Strunnikov A (1997). A direct link between sister chromatid cohesion and chromosome condensation revealed through the analysis of MCD1 in S. cerevisiae. Cell.

[31] Bloom MS, Koshland D, Guacci V (2018). Cohesin function in cohesion, condensation, and DNA repair is regulated by Wpl1p via a common mechanism in Saccharomyces cerevisiae. Genetics.

[32] Yi Q, Chen Q, Yan H, Zhang M, Liang C, Xiang X (2019). Aurora B kinase activity-dependent and -independent functions of the chromosomal passenger complex in regulating sister chromatid cohesion. J Biol Chem.

[33] Hagstrom KA, Holmes VF, Cozzarelli NR, Meyer BJ (2002). C. elegans condensin promotes mitotic chromosome architecture, centromere organization, and sister chromatid segregation during mitosis and meiosis. Genes Dev.

[34] Hauf S, Cole RW, LaTerra S, Zimmer C, Schnapp G, Walter R (2003). The small molecule Hesperadin reveals a role for Aurora B in correcting kinetochore-microtubule attachment and in maintaining the spindle assembly checkpoint. J Cell Biol.

[35] Cimini D, Wan X, Hirel CB, Salmon ED (2006). Aurora kinase promotes turnover of kinetochore microtubules to reduce chromosome segregation errors. Curr Biol.

[36] Ditchfield C, Johnson VL, Tighe A, Ellston R, Haworth C, Johnson T (2003). Aurora B couples chromosome alignment with anaphase by targeting BubR1, Mad2, and Cenp-E to kinetochores. J Cell Biol.

[37] O’Connor A, Maffini S, Rainey MD, Kaczmarczyk A, Gaboriau D, Musacchio A (2015). Requirement for PLK1 kinase activity in the maintenance of a robust spindle assembly checkpoint. Biol Open.

[38] Saurin AT, van der Waal MS, Medema RH, Lens SM, Kops GJ (2011). Aurora B potentiates Mps1 activation to ensure rapid checkpoint establishment at the onset of mitosis. Nat Commun.

[39] Shao H, Huang Y, Zhang L, Yuan K, Chu Y, Dou Z (2015). Spatiotemporal dynamics of Aurora B-PLK1-MCAK signaling axis orchestrates kinetochore bi-orientation and faithful chromosome segregation. Sci Rep.

[40] Santaguida S, Amon A (2015). Short- and long-term effects of chromosome mis-segregation and aneuploidy. Nat Rev Mol Cell Biol.

[41] Thompson SL, Compton DA (2011). Chromosome missegregation in human cells arises through specific types of kinetochore-microtubule attachment errors. Proc Natl Acad Sci USA.

[42] Gregan J, Polakova S, Zhang L, Tolic-Norrelykke IM, Cimini D (2011). Merotelic kinetochore attachment: Causes and effects. Trends Cell Biol.

[43] Cimini D, Howell B, Maddox P, Khodjakov A, Degrassi F, Salmon ED (2001). Merotelic kinetochore orientation is a major mechanism of aneuploidy in mitotic mammalian tissue cells. J Cell Biol.

[44] Ferreira LT, Orr B, Rajendraprasad G, Pereira AJ, Lemos C, Lima JT, et al. alpha-Tubulin detyrosination impairs mitotic error correction by suppressing MCAK centromeric activity. J Cell Biol. 2020;219:e201910064. 10.1083/jcb.201910064 PMC7147099 32328631

[45] Liu Y, Robinson D. Recent advances in cytokinesis: understanding the molecular underpinnings. F1000Res. 2018;7:F1000. 10.12688/f1000research.16502.1 PMC6259594 30542616

[46] Pike T, Brownlow N, Kjaer S, Carlton J, Parker PJ (2016). PKCvarepsilon switches Aurora B specificity to exit the abscission checkpoint. Nat Commun.

[47] Waizenegger IC, Hauf S, Meinke A, Peters JM (2000). Two distinct pathways remove mammalian cohesin from chromosome arms in prophase and from centromeres in anaphase. Cell.

[48] Hauf S, Waizenegger IC, Peters JM (2001). Cohesin cleavage by separase required for anaphase and cytokinesis in human cells. Science.

[49] Bourmoum M, Charles R, Claing A. ARF6 protects sister chromatid cohesion to ensure the formation of stable kinetochore-microtubule attachments. J Cell Sci. 2018;131:jcs216598. 10.1242/jcs.216598 29724911

[50] Diaz-Martinez LA, Gimenez-Abian JF, Clarke DJ (2007). Cohesin is dispensable for centromere cohesion in human cells. PLoS One.

[51] Silva RD, Mirkovic M, Guilgur LG, Rathore OS, Martinho RG, Oliveira RA (2018). Absence of the spindle assembly checkpoint restores mitotic fidelity upon loss of sister chromatid cohesion. Curr Biol.

[52] Mirkovic M, Hutter LH, Novak B, Oliveira RA (2015). Premature sister chromatid separation is poorly detected by the spindle assembly checkpoint as a result of system-level feedback. Cell Rep.

[53] Hirano T. Chromosome dynamics during mitosis. Cold Spring Harb Perspect Biol. 2015;7:a015792. 10.1101/cshperspect.a015792 PMC4448609 25722466

[54] Elbatsh AMO, Kim E, Eeftens JM, Raaijmakers JA, van der Weide RH, Garcia-Nieto A (2019). Distinct roles for condensin’s two ATPase sites in chromosome condensation. Mol Cell.

[55] Hirano T (2012). Condensins: Universal organizers of chromosomes with diverse functions. Genes Dev.

[56] Shintomi K, Hirano T (2011). The relative ratio of condensin I to II determines chromosome shapes. Genes Dev.

[57] Green LC, Kalitsis P, Chang TM, Cipetic M, Kim JH, Marshall O (2012). Contrasting roles of condensin I and condensin II in mitotic chromosome formation. J Cell Sci.

[58] Gerlich D, Hirota T, Koch B, Peters JM, Ellenberg J (2006). Condensin I stabilizes chromosomes mechanically through a dynamic interaction in live cells. Curr Biol.

[59] Tanno Y, Kitajima TS, Honda T, Ando Y, Ishiguro K, Watanabe Y (2010). Phosphorylation of mammalian Sgo2 by Aurora B recruits PP2A and MCAK to centromeres. Genes Dev.

[60] Lipp JJ, Hirota T, Poser I, Peters JM (2007). Aurora B controls the association of condensin I but not condensin II with mitotic chromosomes. J Cell Sci.

[61] Tada K, Susumu H, Sakuno T, Watanabe Y (2011). Condensin association with histone H2A shapes mitotic chromosomes. Nature.

[62] Funabiki H (2019). Correcting aberrant kinetochore microtubule attachments: A hidden regulation of Aurora B on microtubules. Curr Opin Cell Biol.

[63] Krenn V, Musacchio A (2015). The aurora B kinase in chromosome Bi-orientation and spindle checkpoint signaling. Front Oncol.

[64] Joukov V, De Nicolo A. Aurora-PLK1 cascades as key signaling modules in the regulation of mitosis. Sci Signal. 2018;11:eaar4195. 10.1126/scisignal.aar4195 30108183

[65] Kim W, Ryu J, Kim JE. CCAR2/DBC1 and Hsp60 positively regulate expression of survivin in neuroblastoma cells. Int J Mol Sci. 2019;20:131. 10.3390/ijms20010131 PMC6337645 30609639

[66] Choi M, Kim W, Cheon MG, Lee CW, Kim JE (2015). Polo-like kinase 1 inhibitor BI2536 causes mitotic catastrophe following activation of the spindle assembly checkpoint in non-small cell lung cancer cells. Cancer Lett.

[67] Drpic D, Almeida AC, Aguiar P, Renda F, Damas J, Lewin HA (2018). Chromosome segregation is biased by kinetochore size. Curr Biol.

[68] Braga LG, Prifti DK, Garand C, Saini PK, Elowe S. A quantitative and semi-automated method for determining misaligned and lagging chromosomes during mitosis. Mol Biol Cell. 2020;32:mbcE20090585. 10.1091/mbc.E20-09-0585 PMC8108530 33085580

[69] Howe B, Umrigar A, Tsien F. Chromosome preparation from cultured cells. J Vis Exp. 2014;83:e50203. 10.3791/50203 PMC4091199 24513647

