# Supplementary material for: CCAR2 controls mitotic progression through spatiotemporal regulation of Aurora B
Source: Cell Death Dis. 2022 Jun 7;13(6):534. doi: 10.1038/s41419-022-04990-8 (PMC9174277; doi:10.1038/s41419-022-04990-8)
Supplement: Supplementary file 1 — Supplementary Information [file 41419_2022_4990_MOESM1_ESM.pdf]

SUPPLEMENTARY INFORMATION

CCAR2 controls mitotic progression through spatiotemporal regulation of Aurora B

Jaewook Ryu [1], Ja-Eun Kim [1,2,*]

[1]Department of Biomedical Science, Graduate School, Kyung Hee University, Seoul 02447, Korea
[2]Department of Pharmacology, School of Medicine, Kyung Hee University, Seoul 02447, Korea

* Corresponding author. Department of Pharmacology, School of Medicine, Kyung Hee University, 26, Kyunghee-daero, Dongdaemun-Gu, Seoul 02447, Republic of Korea.
email: jekim@khu.ac.kr

SUPPLEMENTARY MATERIALS AND METHODS

Annexin V/Propidium iodide (PI) staining

The FITC Annexin V apoptosis detection kit I (BD Pharmingen; 556547) was used to detect apoptotic cells. Briefly, cells were washed twice with PBS, and then stained for 15 min at room temperature in the dark with Annexin V-FITC and PI in binding buffer. Early apoptotic (Annexin V-positive, PI-negative) and late (Annexin V-positive and PI-positive) apoptotic cells were counted using a flow cytometer [1].

Analysis of cell cycle phase using a flow cytometer

Each cell cycle phase was assessed by DNA content using propidium iodide (PI). S phase and mitosis was identified by staining 5-bromo-2'deoxyuridine (BrdU)-incorporated DNA and phosphorylated histone H3 ser10 (H3-pS10), respectively. Cells were suspended in PBS and then 100% ethanol was added to be the final concentration of 70% ethanol while gently vortexing. The fixed cells were permeabilized with 0.25% Triton X-100 in PBS on ice for 15 min. The cells were incubated with anti-H3-pS10 (Merck; 06-570) antibody for 2 h, and then incubated with secondary antibody at room temperature in the dark for 1 hr. For 5-bromo-2'deoxyuridine (BrdU) staining, cells were pulse-labeled with 30 μM BrdU (Sigma; B5002) for incorporation into DNA for 30 min at 37 °C in 5% CO_2 incubator. After fixation as described above, DNA was denatured by immersion with 2 N HCl-0.5% Triton X-100 for 30 minutes at room temperature. After washing with PBS, pellet was resuspended in 0.1 M $Na_2B_4O_7$ (pH 8.5). The cells washed with PBS were resuspended in PBS with 0.5% Tween 20 and 1% BSA. The cells were incubated with anti-BrdU antibody antibody (BD Pharmingen; 555627) for 2 h, and then incubated with secondary antibody at room temperature in the dark for 1 hr. After staining with anti-H3-pS10 or anti-BrdU, cells were incubated with RNase A at 37 °C for 30 min and then PI at 37 °C in the dark for another 30 min. Cell cycle phases were analyzed by flow cytometry [1].

Reverse transcription-polymerase chain reaction (RT-PCR)

Total RNA was isolated using TRIzol (Invitrogen; 15596026), and cDNA was synthesized using PrimeScript™ reverse transcriptase (Takara; 2680A). cDNA is used as a template for semi-quantitative PCR. The PCR product was visualized by agarose gel electrophoresis. The sequences of each forward (F) and reverse (R) primer used for PCR were as follows: CCAR2-F, CAAACATCCCACACACTTCAC; CCAR2-R, GACCTGGATCCGGCTTGGATG; Aurora B-F, CCTTGGACCCCAGCTCTCCTC; Aurora B-R, TCTCCCGAGCCAAGTACACG; PLK1-F, GGTTTTCGATTGCTCCCAGC; PLK1-R, AGCTGATACCCAAGGCCGTA; β-actin-F, GCTCGTCGTCGACAACGGCT; and β-actin-R, CAAACATGATCTGGGTCATCTTCTC.

Pull-down assay

HEK293T cells were transfected with S- and FLAG-tagged CCAR2 and 3×Myc-tagged Aurora B. HEK293T cell lysates were used for pull-down using S-protein agarose (Merck; 69704). Western blotting was performed following a routine protocol.

SUPPLEMENTARY RESULTS

CCAR2 deficiency does not induce cell death and cell cycle arrest

Because clonogenic assay cannot distinguish between cell death and proliferation defect, flow cytometry was used to detect cell death and to assess cell cycle phases. First, populations of cells undergoing apoptosis and necrosis was evaluated by Annexin V and PI staining, respectively. While BI2536-treated cells (used as a control group) contained a significant apoptotic population, siCCAR2 cells did not show significant apoptosis or necrosis (Supplementary Fig. 1A). This suggests that low survival of siCCAR2 cells results from a proliferation defect, not cell death. Next, the percentage of cells in each cell cycle phase was determined by measuring PI-stained DNA content. Simultaneously, cells undergoing replication and mitosis were examined by measuring BrdU pulse-labeled and H3-pS10-associated DNA content, respectively. Unexpectedly, the overall portion of cells in S phase slightly increased in siCCAR2 cells although this change was not significant (Supplementary Fig. 1B). The number of BrdU-positive cells was not significantly different between siCon and siCCAR2 cells, but also showed a tendency to increase in siCCAR2 cells (Supplementary Fig. 1C). This increasing tendency in S phase might be related to cohesion establishment (discussed in the Results). In addition, the number of cells containing H3-pS10 positivity, a marker for cell division, was similar in siCon and siCCAR2 cells (Supplementary Fig. 1D). The similar duration from prophase to telophase (Fig. 2D, left panel) in both cells is consistent with the data of the similar number of H3-pS10-positive cells (Supplementary Fig. 1D). As shown in immunocytochemistry, H3-pS10 is present from prophase to telophase but absent in cytokinesis (Supplementary Fig. 2A). Taken together, considering a low cell survival in siCCAR2 cells (Fig. 1B), an increase in S phase population does not mean active proliferation. Instead, this indicates that CCAR2 deficiency disturbs cell proliferation.

CCAR2 is essential for faithful mitotic progression

HeLa cancer cells, as well as IMR-90 and WI-38 normal cells, were also transfected with two different CCAR2 siRNAs (Supplementary Fig. 3A). The effect of CCAR2 deficiency on multilobulation was common in all cells (Supplementary Fig. 3B). The duration of each mitotic phase was indirectly assessed

by staining chromosomes and microtubules, and then counting the number of cells in each phase. A significant decrease in cell numbers in prometaphase, and an increase in numbers in cytokinesis, was detected in A549, HeLa, IMR-90, and WI-38 cells (Supplementary Fig. 3C).

CCAR2 regulates the binding with Aurora B

To rule out the possibility that CCAR2 deficiency downregulates expression of Aurora B, resulting in less recruitment, we measured the level of mRNA and protein in siCCAR2 cells. CCAR2 did not affect expression of Aurora B in A549 cells (Fig. 4D, Supplementary Fig. 4A). The expression of Aurora B protein was similarly observed in HeLa, WI-38, and IMR-90 cells (Supplementary Fig. 3A). To determine whether CCAR2 interacts with Aurora B, we examined the ectopic binding of CCAR2 and Aurora B in HEK293T cells. CCAR2 forms a complex with Aurora B (Supplementary Fig. 4B). However, we could not clarify the endogenous interaction between them because a heavy chain from antibody used for immunoprecipitation masks the size of Aurora B.

CCAR2 is required for activation of PLK1 in early mitosis

The activity of PLK1, which is recruited to kinetochore through diverse platforms including CPC components and Aurora B-phosphorylated Mis18α [2], was also determined by measuring the level of PLK1-pT210, a phosphorylated residue by Aurora A and Aurora B [3]. Although the expression of PLK1 protein and mRNA was not affected by CCAR2 deficiency (Fig. 4D and Supplementary Figs. 3A, 4A), the activity and recruitment of PLK1 at the kinetochore was diminished in siCCAR2 cells (Supplementary Fig. 5A, B). Overall, CCAR2 deficiency results in less recruitment and activation of SAC-related proteins as well as Aurora B in kinetochore. However, considering the interaction between CCAR2 and Aurora B, this suggests that CCAR2 mainly controls Aurora B-dependent SAC activation.

CCAR2 deficiency leads to formation of lagging chromosomes

We found an increase in the number of lagging chromosome-containing siCCAR2 cells in A549 cells (Fig. 6A). This phenomenon was confirmed in HeLa, IMR-90, and WI-38 cells (Supplementary Fig. 6A).

CCAR2 does not regulate central spindle assembly

Assembly of an anti-parallel bundle of microtubules to the midzone during anaphase was not affected by CCAR2 deficiency (Supplementary Fig. 7A). Considering that the duration of anaphase and telophase was not prolonged in siCCAR2 cells, formation of the central spindles was not affected although a defect in ingression might produce lagging chromosomes. In addition, enrichment of microtubules at midbody during

cytokinesis was not affected by CCAR2 deficiency (Supplementary Fig. 7B).

REFERENCES

1. Choi M, Kim W, Cheon MG, Lee CW, Kim JE. Polo-like kinase 1 inhibitor BI2536 causes mitotic catastrophe following activation of the spindle assembly checkpoint in non-small cell lung cancer cells. *Cancer Lett* 357, 591-601 (2015)

2. Singh P, Pesenti ME, Maffini S, Carmignani S, Hedtfeld M, Petrovic A, *et al.* BUB1 and CENP-U, Primed by CDK1, Are the Main PLK1 Kinetochore Receptors in Mitosis. *Mol Cell* 81, 67-87 e69 (2021)

3. Shao H, Huang Y, Zhang L, Yuan K, Chu Y, Dou Z, *et al.* Spatiotemporal dynamics of Aurora B-PLK1-MCAK signaling axis orchestrates kinetochore bi-orientation and faithful chromosome segregation. *Sci Rep* 5, 12204 (2015)

SUPPLEMENTARY FIGURES

Supplementary Fig. 1.

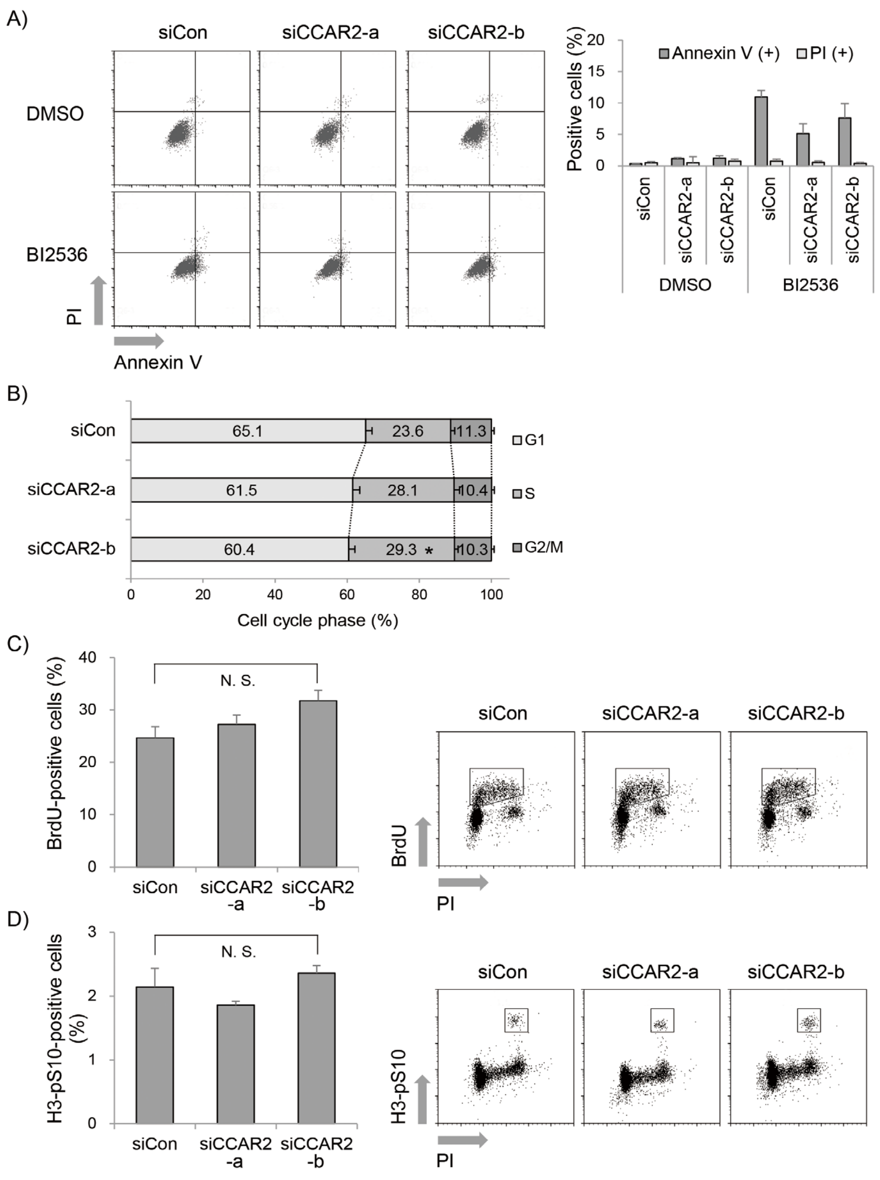

Supplementary Figure 1. CCAR2 deficiency does not induce cell death and cell cycle arrest.

(A-D) A549 cells were transfected with two different siRNA targeting CCAR2. Flow cytometry was done 48 h later. (A) Annexin V- and PI-positive cells represent apoptosis and necrosis, respectively. Apoptosis was induced following treatment of cells with 50 nM BI2536 for 40 h. The experiment was repeated independently (N = 3). (B–D) The cells were analyzed using a flow cytometer. (B) DNA content was verified by staining with PI (N = 6 from data of (C) and (D)). (C) The BrdU pulse labeling allows the detection of cells undergoing replication (N = 3). (D) Mitosis was identified by staining against H3-pS10 (N = 3).

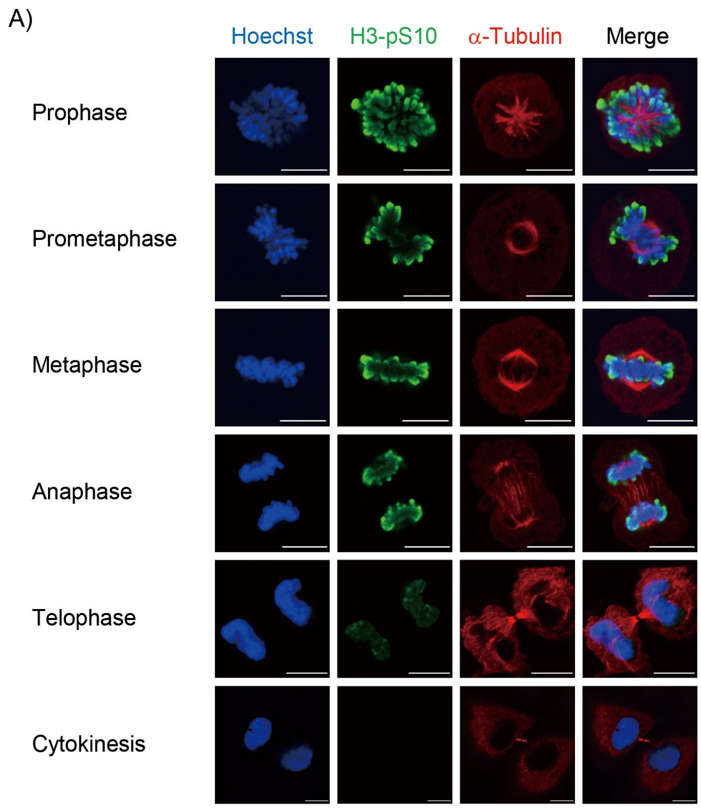

Supplementary Figure 2. H3-pS10 is maintained from prophase to telophase.

(A) Condensed chromosomes and mitotic phases were visualized by staining with Hoechst and anti-H3-pS10 antibody. Scale bar, 10 μm.

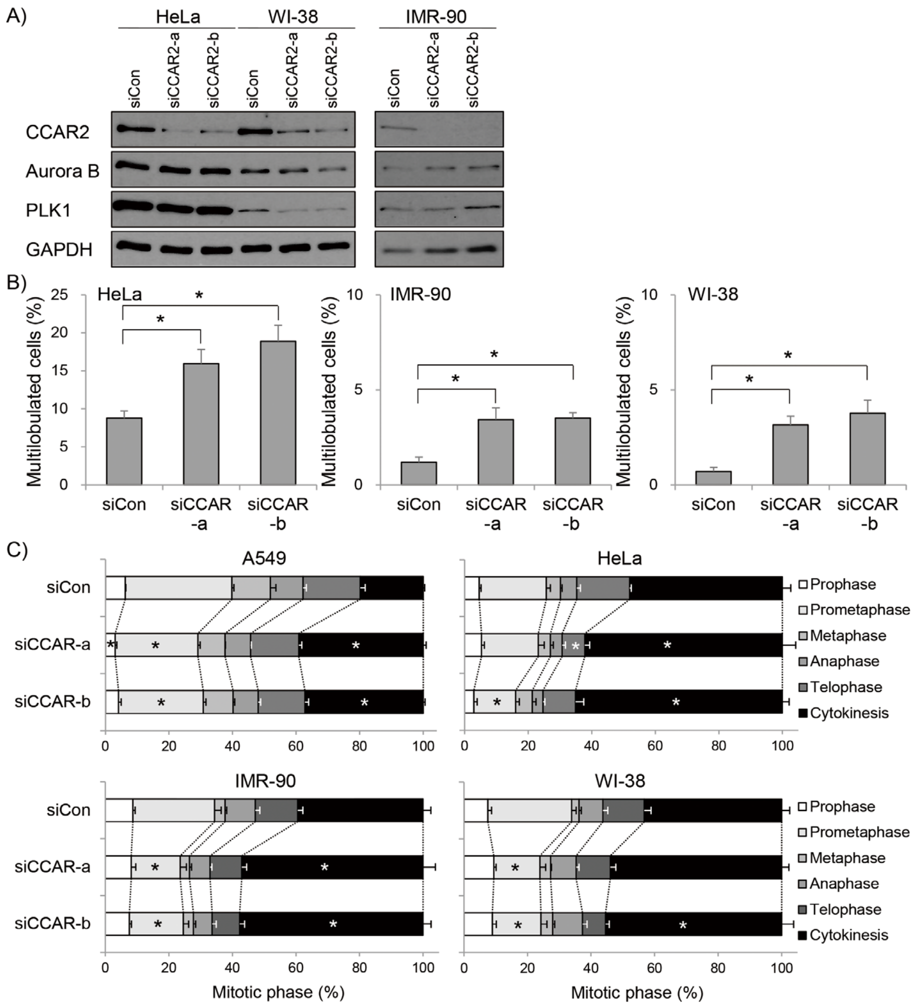

Supplementary Figure 3. CCAR2 deficiency results in aberrant mitotic progression.

A549, HeLa, IMR-90 or WI-38 cells were transfected with two different CCAR2 siRNAs. (A) Expressions of CCAR2 and each protein were verified by western blotting. (B-C) Nuclei, the cytoplasm, and the centrosome were visualized by staining with Hoechst, anti-α-tubulin, and anti-pericentrin antibodies, respectively. (B) The number of cells containing a multilobulated nucleus was counted from out of more than 200 cells per experiment. The experiment was repeated independently (N = 5). The number of interphase cells examined in all experiments is as follows; HeLa – siCon, n = 1777; siCCAR2-a, n = 1812; siCCAR2-b, n = 1725; IMR-90 – siCon, n = 1999; siCCAR2-a, n = 2004; siCCAR2-b, n = 1916; WI-38 – siCon, n = 1962; siCCAR2-a, n = 1909; siCCAR2-b, n = 1803. (C) The number of cells in each mitotic phase was counted based on the status of chromosome condensation and microtubule assembly in more than 100 mitotic cells per experiment. The experiment was repeated independently (N = 5). Data are expressed as the mean ± standard error of the mean (SEM). *$p < 0.05$, significantly different between multiple groups (one-way ANOVA).

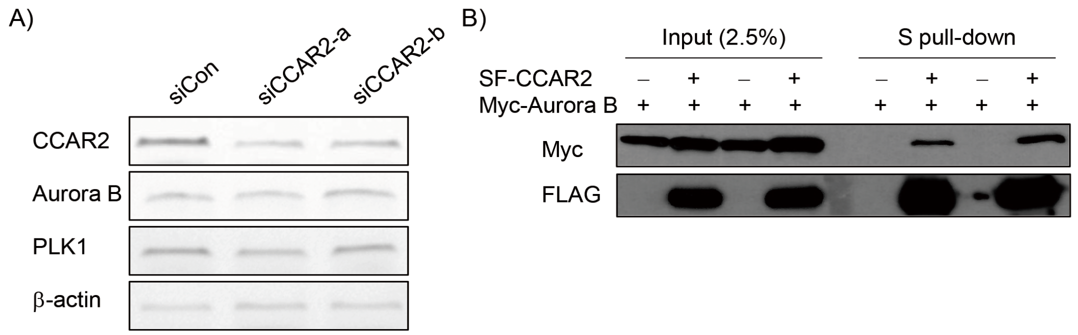

Supplementary Figure 4. CCAR2 does not affect the expression of Aurora B mRNA, but binds with Aurora B.

(A) A549 cells were transfected with two different CCAR2 siRNAs. The expression of each mRNA was validated by semi-quantitative RT-PCR. (B) HEK293T cells were transfected with S/FLAG(SF)-CCAR2 and Myc-Aurora B. The interaction between overexpressed proteins was examined in a pull-down assay using S-agarose beads, followed by western blotting.

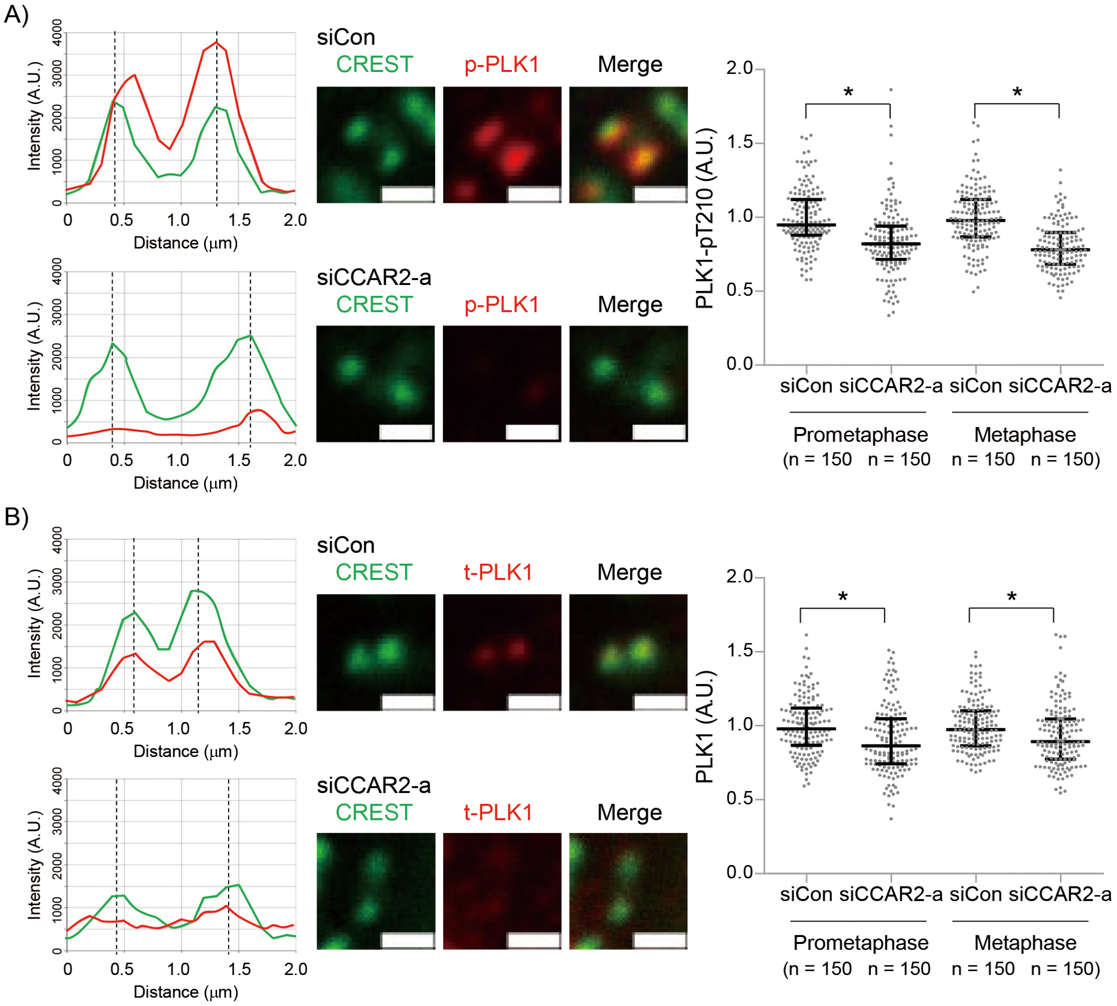

Supplementary Figure 5. CCAR2 deficiency induces inactivation of PLK1 at prometaphase and metaphase.

(A-B) A549 cells were transfected with siRNA-a targeting CCAR2. Asynchronous cells were stained with Hoechst, CREST, and anti-PLK1-pT210 or anti-PLK1 antibodies. Fluorescence intensity of kinetochore-associated PLK1-pT210 (A) and PLK1 (lower panel) in siCCAR2-a cells was normalized to that of siCon cells at prometaphase and metaphase, and presented as a dot plot. In total, 150 kinetochores from 50 cells were examined in five separate experiments (N = 5, n = 150). The first and third bars are the 25th and 75th percentiles, respectively, and the second bar is the median. *$p < 0.05$, significantly different from corresponding siCon cells (two-sided unpaired Student's t-test); A.U., arbitrary units; scale bar, 1 μm.

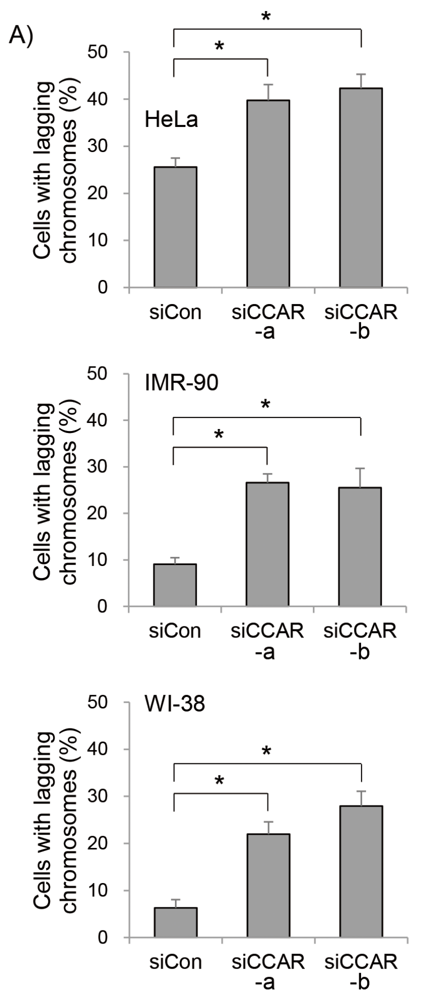

Supplementary Figure 6. CCAR2 deficiency induces lagging chromosomes.

(A) HeLa, IMR-90 or WI-38 cells were transfected with two different CCAR2 siRNAs. Asynchronous cells were stained with Hoechst, anti-α-tubulin, and anti-pericentrin antibodies. The number of cells containing lagging chromosomes at anaphase was counted in five separate experiments (N = 5). The number of anaphase examined in all experiments was as follows; HeLa - siCon, n = 88; siCCAR2-a, n = 67; siCCAR2-b, n = 67; IMR-90 - siCon, n = 78; siCCAR2-a, n = 46; siCCAR2-b, n = 53; WI-38 - siCon, n = 63; siCCAR2-a, n = 53; siCCAR2-b, n = 57. Data are expressed as the mean ± SEM. *$p < 0.05$, significantly different between multiple groups (one-way ANOVA).

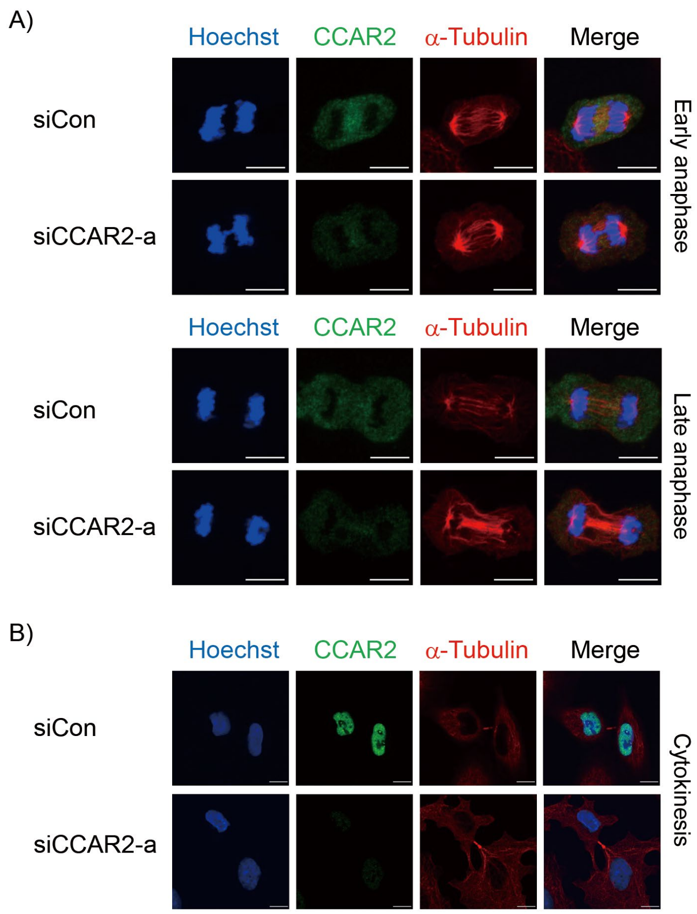

Supplementary Figure 7. CCAR2 deficiency does not affect central spindle assembly.

(A-B) A549 cells were transfected with siRNA-a targeting CCAR2. Asynchronous cells were stained with Hoechst, anti-CCAR2, and anti-α-tubulin antibodies. The localization of α-tubulin at the spindle midzone during anaphase (A), and at midbody during cytokinesis (B), was examined by fluorescence staining.
