# Supplementary figures and images for: CCAR2 controls mitotic progression through spatiotemporal regulation of Aurora B
Source: Cell Death Dis. 2022 Jun 7;13(6):534. doi: 10.1038/s41419-022-04990-8 (PMC9174277; doi:10.1038/s41419-022-04990-8)

Fig. 1A.

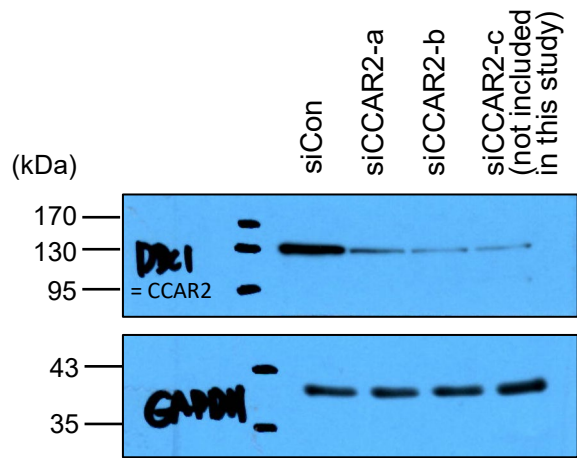

Fig. 1C.

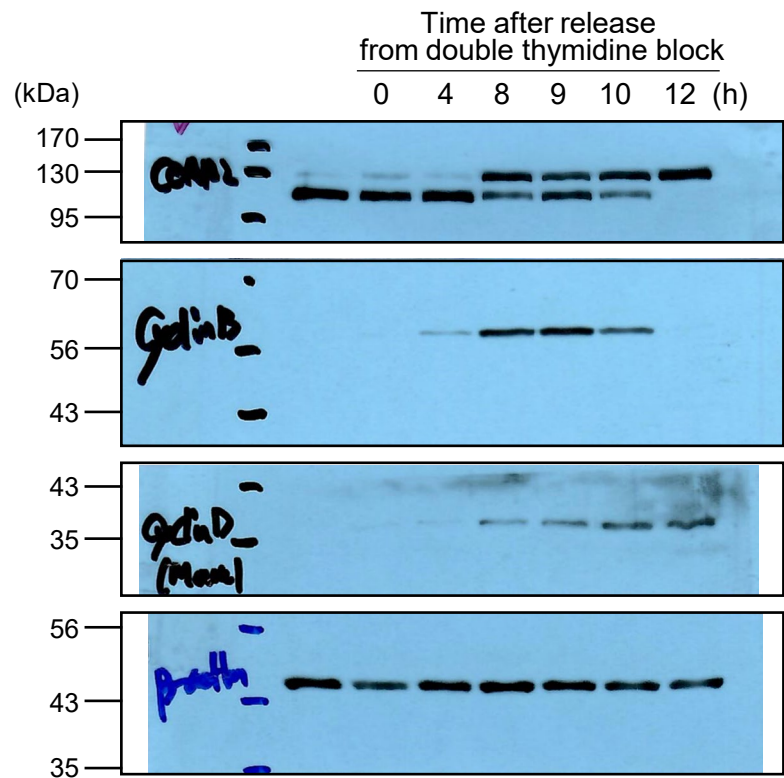

Fig. 4C.

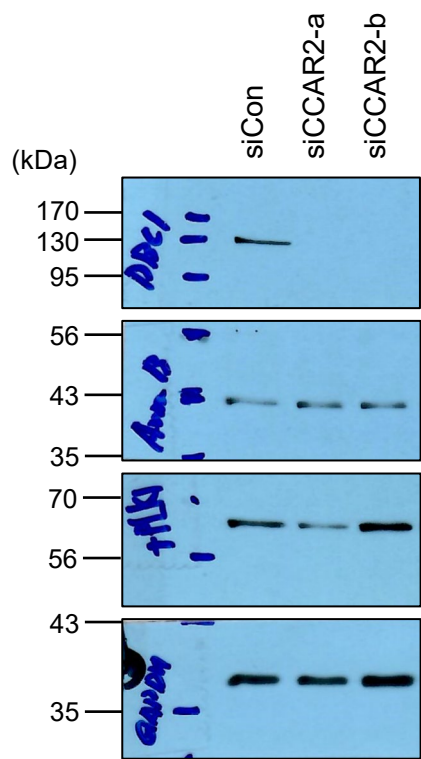

Fig. 5C.

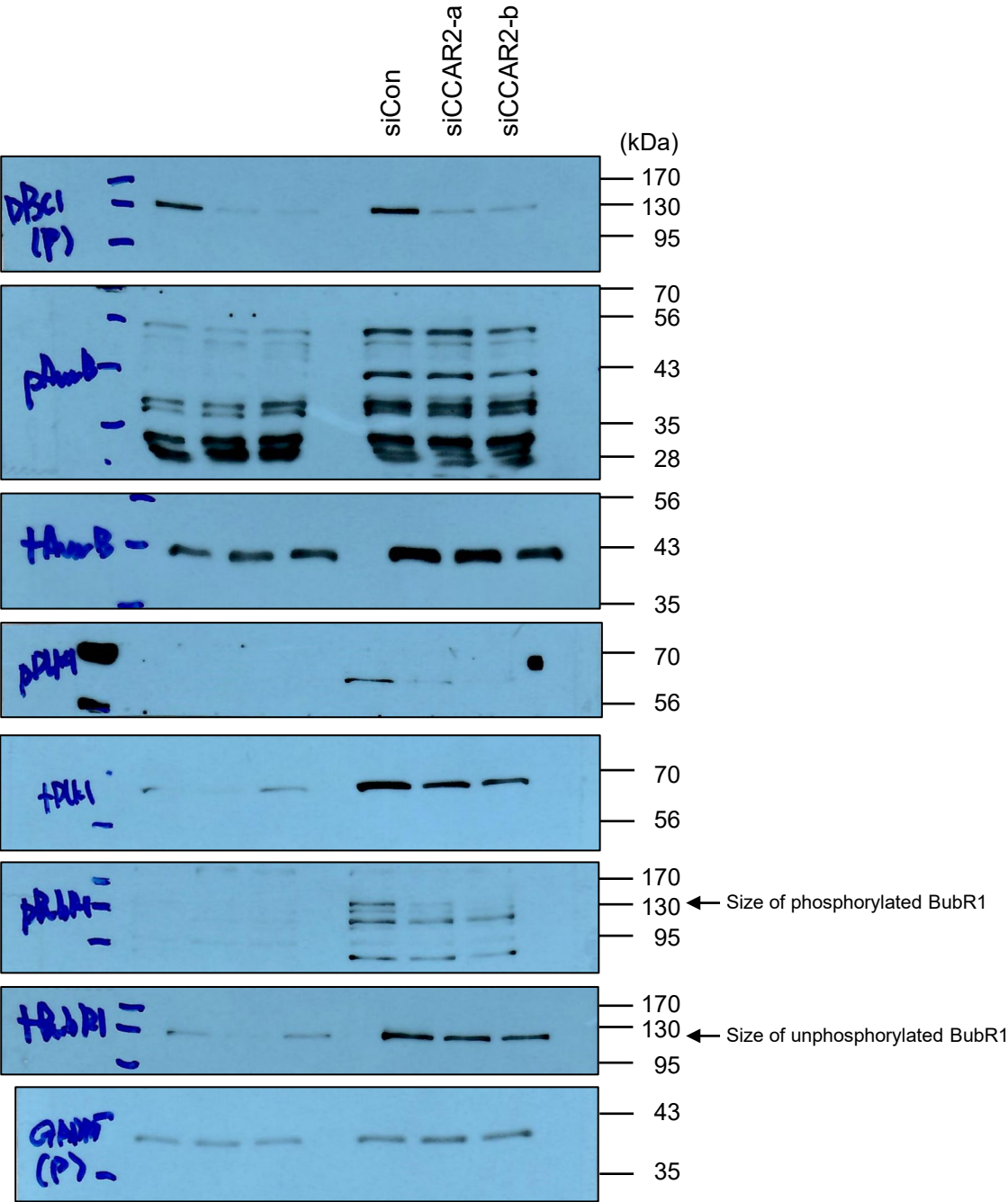

**Fig. 8A.**

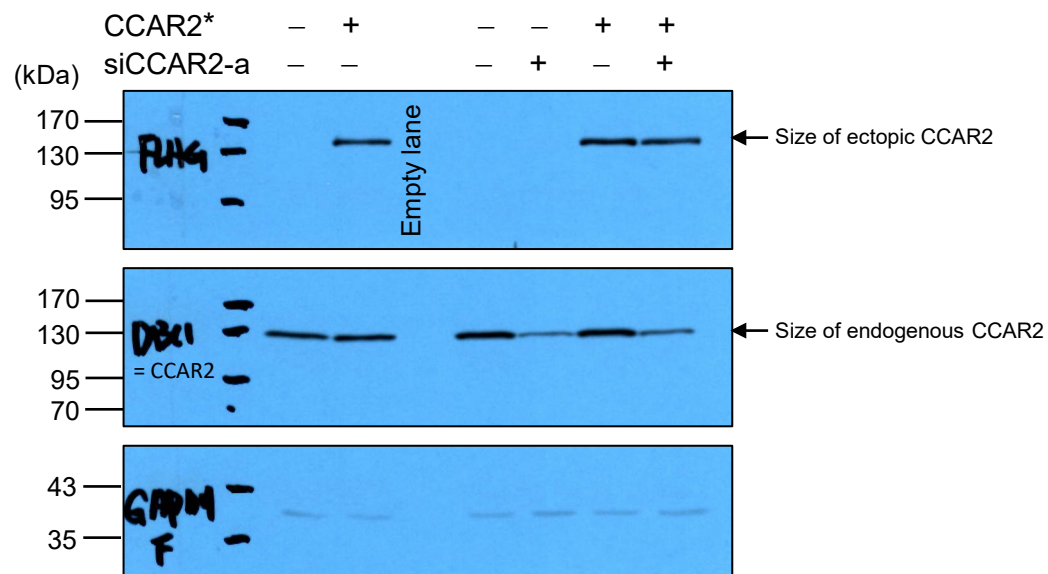

Supplementary Fig. 3A.

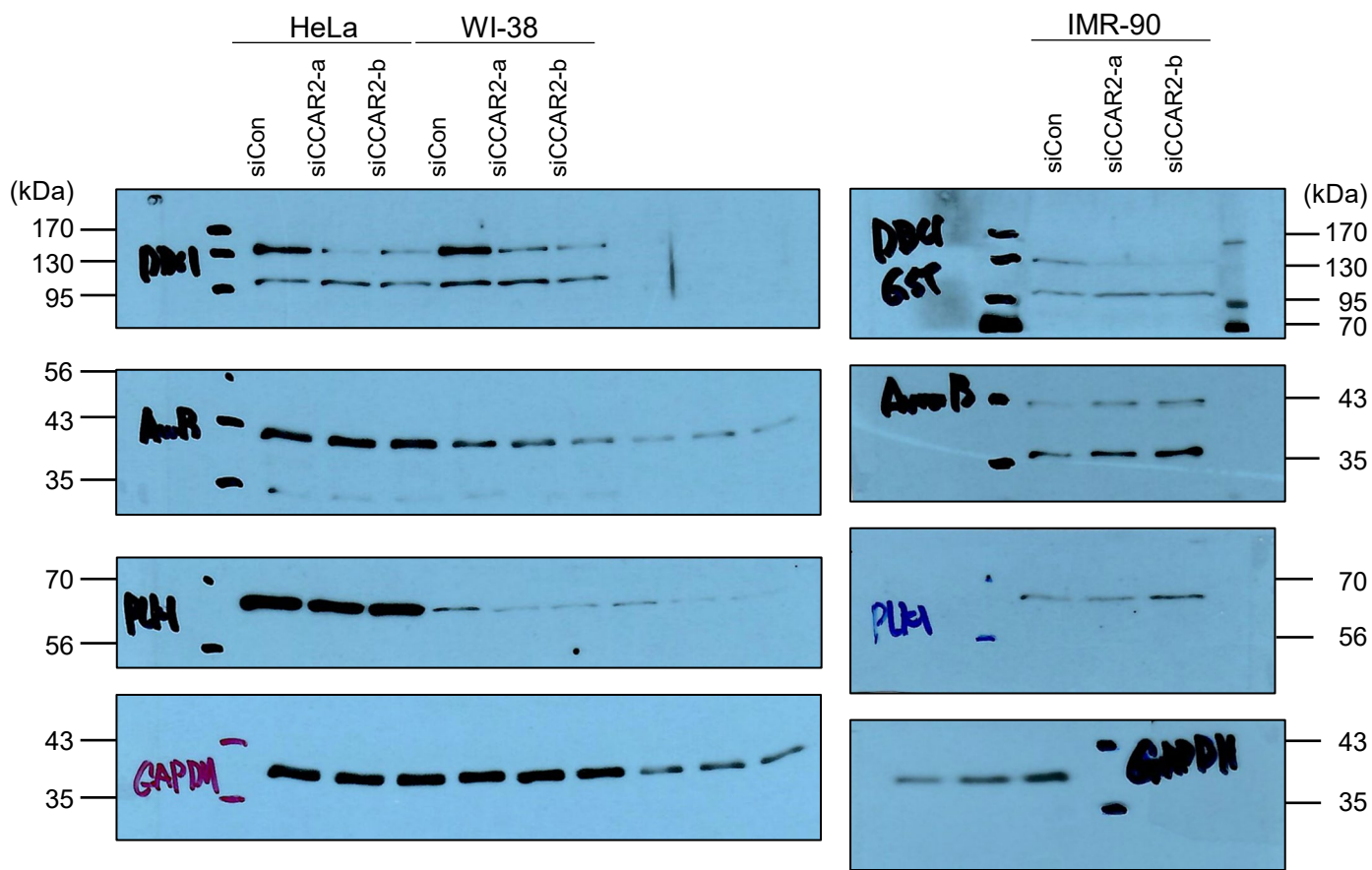

Supplementary Fig. 4A.

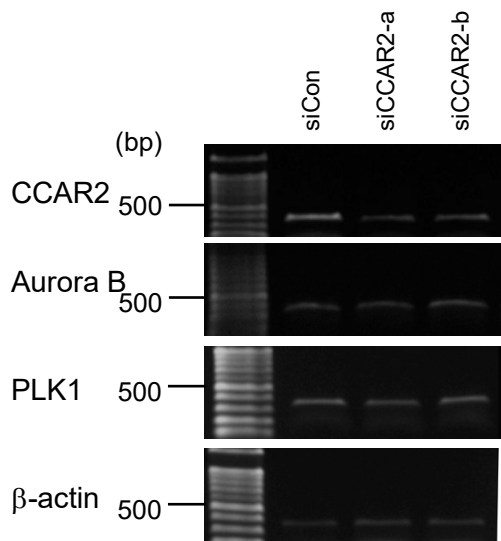

Supplementary Fig. 4B

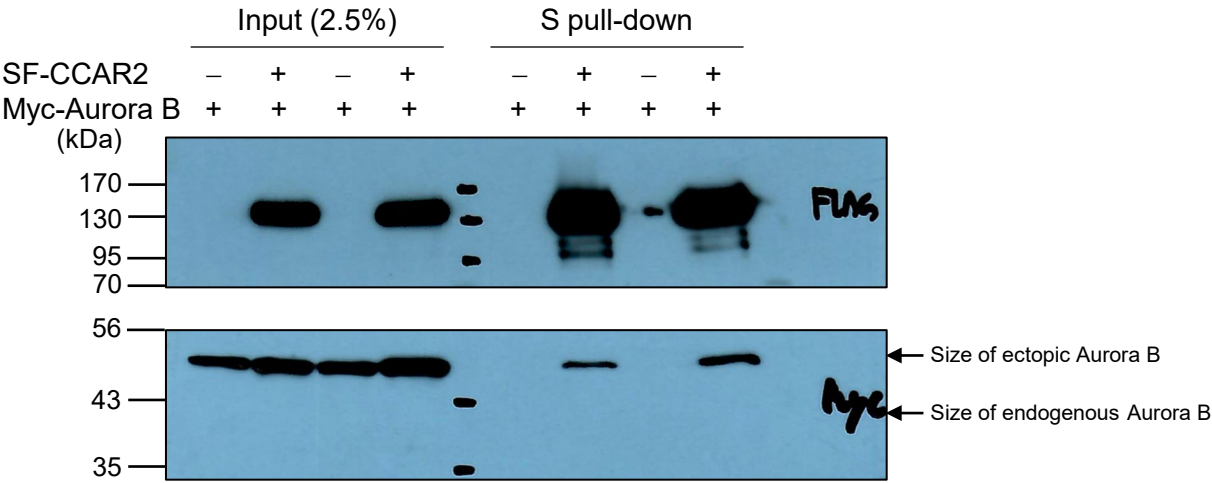

Supplement: Supplementary file 2 — Original Data File [file 41419_2022_4990_MOESM2_ESM.pdf]
